# Supplementary material for: Ret kinase-mediated mechanical induction of colon stem cells by tumor growth pressure stimulates cancer progression in vivo
Source: Commun Biol. 2022 Feb 17;5:137. doi: 10.1038/s42003-022-03079-4 (PMC8854631; doi:10.1038/s42003-022-03079-4)
Supplement: Supplementary file 2 — Supplementary Information [file 42003_2022_3079_MOESM2_ESM.pdf]

Supplementary Notes and Supplementary Figures

Supplementary Notes

Supplementary Note 1

a- *Ex-vivo*, mice colonic pulsatile strains of 2s period are found to have an amplitude of several hundreds of microns, equivalent to the amplitude of ~ 1min period movements[1]. On the other hand, pressure pulsations associated to the 1min periodic movement were measured to be on the order of ~1kPa[2]. Even though pressure measurement methodology was revealed to remove high frequency movements (by insertion of a basal loading pressure into the gut required for instrumental pressure measurements), so preventing direct measurement its motor pressure pulsations[3], the similitude between the amplitudes of the ~ 1min low frequency and 2s high frequency period within the same tissue indicates a ~ 1kPa similar pressure amplitude for both frequency movements.

b- Note that the mechanical effect of WIN on Lgr5 expression can intrinsically not be separated from any effect that would potentially be biochemical in nature. The rescue of physiological levels of Lgr5 in response to the rescue of the pulsatile pressure mechanical stresses by magnetic means (Fig.1e-g) of the following-up experiments confirms a mechanical effect.

Supplementary Note 2

a- UML were previously found to have extravasated in the conjunctive tissue of the colon, with co-localization with Vimentin positive mesenchymal cells, 30 min after injection, and to be stable at least 1 week, in the presence of the external stabilizing magnet[4]. Here we further characterize that this feature is stable at least 3.5 months after the removal of the external stabilizing magnet applied for 30min (Supplementary Fig. 2a,b). Note that the presence of the stabilizing magnet was required for the presence of injected UML into the conjunctive tissue, as observed by the absence of UML after injection without magnet (Ctrl) compared to with magnet (5d) in the conjunctive tissue after 5 days (Supplementary Fig. 2a,b). How the stabilizing magnet promotes extravasation of UML circulating into micro-vessels through localized magnetic forces application is not known and opens to future investigations.

b- Note that the 6% of the blood cell type that showed an internalisation of UML could not be distinguished with the flow cytometry methodology used, due to the limitation of the amount of biological material by condition. In addition, the full wash-out of UML from both the blood and these cells between 3h and 24h is in line with the dynamics of 16h of degradation of the UML in blood vessels[5].

Supplementary Note 3

a-To generate force application in a pulsatile regime, we used a 2D network of 1T permanent magnets of 2cm every 2cm (Supplementary Fig3a, see Methods), leading to predicted and measured magnetic fields B^2 of 10^{-3}, 6.10^{-4} and 4.10^{-4} at 2cm, 2.5cm and 3cm, respectively (Supplementary Fig.3b up). This set-up leads to relatively uniform gradients of the square of magnetic fields on the order of $0.06T^2/m$, at 2.5cm of the top of the 2cm size magnets (Supplementary Fig.3b middle). Indeed, this gradient will be on the order of $0.07T^2/m$ on the top of individual magnets in which the z component only counts (Supplementary Fig.3b middle), and in the order of $0.07T^2/m$ between magnets at which x and y components cumulate (Supplementary Fig.3b middle). Maximal magnetic fields B are on the order of 30mT inside the network (Supplementary Fig.3a). UML superparamagnetic particles are magnetized with a moment **m** proportional to the magnetic field $\mathbf{m} \alpha \mathbf{B}$[6]. Therefore, the magnetic force components **Grad**(**m**.**B**) applied to the magnetized tissue will be proportional to these

gradients of square magnetic field **Grad**(B^2). Mice cages were thus positioned in such a way mice colon are at 2.5cm of distance of magnets when plan position is up, with the plan oscillating between 22 cm and 2.5 cm with a 2s period of time (Fig. 1e and Video 3).

The 0.07 T^2/m value is equivalent to the 0.07 T^2/m value produced on the colon at 7mm of distance by the permanent under-skin 1.4T small magnet of 3mm (Supplementary Fig.3b down) already demonstrated to produce a 1kPa pressure in the colon[4]. The device thus produces a magnetically induced pressure pulsation of 2s period and of 1kPa amplitude.

b- Note that the movement induced is not expected to be propagative like natural pulsatile ones. However, the movement should rescue the natural physiologically relevant pulsed mechanical stresses locally felt by colonic tissues at crypt and cell scales, in the WIN-treated pulsatile-defective context.

c- The propagative nature of the movement consists in a key filtering element to separate mice respiration movements from pulsatile movements *in vivo*. We thus had to perform the non-propagative magnetically induced movement rescue measurement *ex-vivo,* in agar-gelatin (Fig. 1f). Note that *ex-vivo* in gelatine, spontaneous pulsed movements are impaired with no requirement of WIN.

d- Because the magnetized tissue are conjunctive mesenchymal cells in which epithelial crypts are embedded[4], magnetic forces are generated directly on crypts, from a tissue that is on the order of 10 times thinner than the surrounding visceral muscle tissue of the colon from which comes natural forces of pulsatile movements[7]. Because the force developed from these distinct tissues is proportional to the pressure developed inside, times the tissue surface, the deformation of the overall colon structure by a 1kPa magnetic pressure generated by the conjunctive tissue is on the order of 100 times smaller than the force generated by endogenous 1kPa pressure from the visceral muscles. Which we observe on Fig. 1f versus Fig. 1d.

Supplementary Note 4
In contrast to a permanent application of the 3mm magnet on-skin for 4 hours to one month showing an activation of the pathological signal of pRet (the base of the crypts shows ≥4 pRet positive cells) [4], its application for 30 min, which is sufficient to stabilise UML into the colon[4], is not sufficient to trigger the activation of Ret neither at 4h nor at one month (Supplementary Fig.5). And indeed, no rescue was observed in UML-loaded colon crypts of WIN-treated mice in the absence of pulsed stress (Fig. 2a,b).

Supplementary Note 5
Importantly, we checked that the other targets of Vande, EGFR and VEGFR2[8] were not spontaneously activated, or mechanically activated by *ex-vivo* and *in-vivo* compression in 4 months mice, indicating that the inhibiting effect of Vande is specific of Ret mechanotransductive activation (Supplementary Fig.6a-e). We additionally checked that Vande treatment does not repress the spontaneous phosphorylation of EGFR and VEGFR2 found in 16 months old APC+/- mice generating sporadic gastric tumors, at concentration used (Supplementary Fig.6f-h). This furthermore shows that, over its 3 targets Ret, EGFR and VEGFR2, Vande specifically represses only Ret activation in the colon at the concentration used that further ensures a specific inhibiting effect of Ret by Vande in all experiments.

Supplementary Note 6
a- Note that the role a small number of mechanically induced Ret positive cells and crypts in Lgr5+ cells expression suggests the existence of a transient dynamical mechanical activation of Ret in a few cells of the 3D crypts, or of few crypts, that could affect all crypts overtime. This hypothesis would be experimentally tested in future work.
b- Detection of the β-cat pathway mechanical activation in immunofluorescence can take several weeks under mechanical stress *in vivo*[4], probably due to the high sensitivity of transcription targets

expression to very low levels of β-cat[9,10], preventing us to monitor the β-cat activation downstream of Ret at the 2 to 5 day time-scale of the present experiment, *in vivo*.

Supplementary Note 7

In contrast to pulsatile stress stimulation in which both the change in the number of Lgr5+ cells per crypt (not all crypts show Lgr5+-GFP in the mosaic mice B6.129P2-Lgr5$^{tm1(cre/ERT2)Cle}$ model controls) and of Lgr5+ crypts per mouse were measured and observed to most sensibly detect effects on Lgr5, the measurement of Lgr5+ cells per crypt only was enough to sensitively detect the effects of the permanent stress more intense stimulation on Lgr5.

Supplementary Note 8

The presence of UML into Vimentin positive cells of the conjunctive mesenchyme is stable for at least 3.5 months (Supplementary Fig.8a,c). This *a priory* excludes its presence in activated macrophages due to any inflammatory response, in which it should be rapidly eliminated and could not be stabilized for months, and indicates that UML are stabilized into the mesenchymal cells of the conjunctive tissue of the colon including fibroblasts. In addition, the presence of UML *per se* (*i.e* in the absence of magnet applied for one month) does not show any inflammatory or fibrotic anatomopathological phenotype[4], excluding any UML-induced inflammation and fibrosis that would participate to the Ret dependent stimulation of SC and hyperproliferation in epithelial cells in addition to mechanical stimulation.
Finally, Ret activation is observed 1 minute only after mechanical stimulation *ex-vivo*, and consistently at the time UML stabilization (30min after injection in the presence of the stabilization magnet) in the mesenchyme *in vivo*[4]. Such one-minute time scale, in line with the mechanotransductive activation of Ret, is too short to activate any inflammatory response that should take at least 4 days[11], or of fibrotic protein expression, protein production taking at least several hours into the colon[12]. No inflammatory or fibrotic response to the presence of UML *per se*, or to magnetic forces applied by the magnet on the UML stabilized into the mesenchymal cells of the conjunctive tissue of the colon, can thus participate to the initiation of the activation of Ret that leads to the stimulation of SC and of hyperproliferation in epithelial cells, in addition to direct mechanical stimulation.

Supplementary Note 9

We used Apc;Lgr5-EGFP and Apc;N1CreERT2 mice older than 4 months presenting colorectal tumors to search for cells positive to CSC markers. To detect such mice, we analyzed by colonoscopy the descending colon of mice using a small-animal colonoscope, to visualize the gut wall and identify abnormalities. We could visualize ACF and also large tumors in the cavity colon of these mice. The larger tumors occupy half of the diameter of the colon and harbor multiples white ACFs. After dissection, we could visualize 2 tumors in the proximal and distal colon and observed multiple tumors in the small intestine (Supplementary Fig.10c). Histological sections reveal adenocarcinoma with crypt fusion and hyperplasia.

Supplementary Note 10

The generation of Paneth cells is also interestingly consistent with the increase of Paneth cells observed in a mouse model of dietary-induced sporadic small and large intestinal cancer[13].

Supplementary Note 11

1kPa tumor growth pressure stimulates tumor-initiator ACF formation after 1 month[4] sensitively enough to check the effect of Vande on ACF mechanical induction with a treatment duration no longer than one month. However, the ACF, that are detectable in colonoscopy with a blue coloration, are sporadically induced, and cannot be localized by eye in post-surgery explants, which prevents their targeted observation by histological cuts.

Supplementary Note 12

a- The Ret positive cells are found into the Lgr5 expressing domain of the crypts bottom, as well as slightly upper. Note that the role of such a small number of mechanically induced Ret positive cells and

crypts in Lgr5+ cells expression suggests the existence of a transient dynamical mechanical activation of Ret in a few cells of the 3D crypts, or of few crypts, that would affect all crypts overtime. A hypothesis to be experimentally tested in future work.

b- Other mechanical strains, such as breathing or motion movements may also mechanically affect the colon and add to 2s spontaneous pulsatile stresses, and thus to stem cells rate maintenance by mechanotransductive signalling. However, breathing movements, which amplitude compares to colon pulsatile movements (see Video 1), operate in a completely different spectral regime (allowing for efficient filtering in our analysis), and involve a global translation movement of the colon rather than local mechanical deformations of the colon. In addition, these breathing-induced translational movements involve the proximal part of the colon (closer to the thorax, left half of the colon in Videos1,2) rather than the distal one in which all biochemical analysis were performed (see Methods). Finally WIN-treated mice did daily walk normally compared to non-treated controls. This thus excludes any interference of breathing movements, or walking movements, in the mechanical stimulation of SC formation in mice and in the present experiments, respectively.

Supplementary Note 13

Note that in a Wnt partially deficient context, an anomalously low number of SC was found to biochemically favor Apc deficient cells fixation in the crypts and to genetically stimulate tumorigenesis in the intestine[14]. We here find that a tumor growth pressure mechanically induces anomalously high level of SC that favors hyperproliferative tumor-initiating ACF formation in the colon. This interestingly indicates the importance of a fine-tuned regulation of SC number to avoid genetically or mechanically induced tumorigenic processes.

Supplementary Note 14

To directly anticipate for pre-clinic and potential future clinic applications, and as the pharmacological approach is here demonstrated to be specific to Ret mechanical activation (Supplementary Fig.6), pharmacological tools were chosen to synergically address both the pulsatile underlying origin and treatment potentialities of mechanotransductive tumor progression (Fig. 6 ,7), based on chemical inhibition of Ret activation. These tools can thus be used in future preclinic studies with PDOX implantation, with possible coupling to magnetic pressure stimulation.

Supplementary Table 1

REAGENT or RESOURCE	SUPPLIER	REFERENCE
**Antibodies**		
pY1062 Ret	Santa Cruz Biotechnology	Ref # 20252
pY1062 Ret	Abcam	Ref # ab51103
β-catenin (Clone 14)	BD Biosciences	Ref # 610153
pY654 β-catenin (1B11)	Santa Cruz Biotechnology	Ref # 57533
Ki67	Abcam	Ref # ab15580
GFP	Abcam	Ref # ab13970
CD133	Abcam	Ref # ab1658
Sox2 (D9B8N)	Ozyme	Ref # 23067S
CD44v6	Sigma-Aldrich	Ref # AB2080
Adh1/2	Santa Cruz Biotechnology	Ref # sc-166362
pY1086 EGFR	Thermo Fisher Scientific	Ref # 36-9700
pY1175 VEGFR2 19A10	Cell Signaling	Ref # 2478
Vimentin	Sigma	Ref # HPA001762
CD31	Novus Biologicals	Ref # NB100-2284
Gli-1	R&D Systems – Bio-Techne	Ref # AF3455
RegIV	Thermo Fisher Scientific	Ref # PA575710
Anti-rabbit Alexa 488	Molecular Probes	Ref # A11008
Anti-goat Alexa 488	Jackson ImmunoResearch	Ref # 705-545-147
Anti-chicken Alexa 488	Abcam	Ref # ab50169
Anti-rabbit Alexa 594	Thermo Fisher Scientific	Ref # A21207
Anti-mouse Alexa 594	Jackson ImmunoResearch	Ref # 715-585-151
Anti-goat Alexa 594	Jackson ImmunoResearch	Ref # 705-585-147
Anti-rabbit Alexa 647	Invitrogen	Ref # A31573
Anti-mouse Alexa 647	Invitrogen	Ref # A31571
**Chemicals**		
DPPC	Avanti Polar Lipids	Ref # 850 355C
DSPC	Avanti Polar Lipids	Ref # 850 365C
DSPE-PEG2000	Avanti Polar Lipids	Ref # 880 135
Rhodamine-PE	Avanti Polar Lipids	Ref # 810146C
Diethyl ether	Sigma-Aldrich	Ref # 179272-1L
Chloroform 99,8%	Sigma-Aldrich	Ref # 319988-1L
HEPES hemisodium salt	Sigma-Aldrich	Ref # H9897-10PAK
NaCl	Sigma-Aldrich	Ref # S7653-1KG
Tri-Sodium citrate dihydrate	Supelco	Ref # 1064481000
R(+)-WIN 55,212-2	Sigma-Aldrich	Ref # W102-25MG
Dimethyl sulfoxide (DMSO)	Sigma-Aldrich	Ref # D2650
Tween (R) 80	Siglma-Aldrich	Ref# P1754
Leibovitz's L15 Medium (1X) + GlutaMAX™	Life Technologies	Ref # 31415-029
Fetal calf serum	Invitrogen	Ref # 10108165
Gentamicin (50 mg/ml)	Life Technologies	Ref # 15750-037
Methylene blue	Sigma-Aldrich	Ref # M9140-25G
Cremophor EL®	Merck	Ref # 238470-1SET
Vandetanib ZD6474	Clinisciences	Ref # HY-10260-500mg
Danusertib	Clinisciences	Ref # HY-10179
ProlongGold DAPI	Thermo Fisher Scientific	Ref # P36935
Tissue-Tek® O.C.T. Compound	VWR	Ref # 4583

Dextrose	Sigma	D934-250g
Tamoxifen	MP Biomedicals	Ref # SKU 0215673891
Antigen Retrieval Reagent-Basic	R&D Systems Bio- Techne	Ref# CTS013
Antigen unmasking solution, citrate-based	Vector laboratories	Ref# H3300
Bond Polymer Refine Detection Kit	Leica	Ref # DS9800
**Experimental models**		
C57BL/6J (JAX™ mice strain)	Charles River Laboratories	
$Apc^{+/1638N}$		Fodde et al., 1994
Lgr5-EGFP-ires-creERT2		RRID:IMSR_JAX:008875
Notch1-CreERT2/Rosa26mTmG		Fre et al. 2011
**Magnets**		
DISQUE - NEO50 3X2	S.A.R.L. AIMANTS CALAMIT	Ref # 0009
NdFeB Neo Delta 398/87 20x20x20mm	Binder magnetic	Ref # AI9PARNFB_00087
**Software**		
ImageJ 1.52p	http://imagej.nih.gov/ij	
Pinnacle Studio		Version 15.0
Adobe Illustrator ®		Version 24.0
BioRender	https://biorender.com/	
KaleidaGraph Version 4.5.2	Synergy Software	
GraphPad Prism Version 7.05	© GraphPad Software, Inc.	

Supplementary Figures

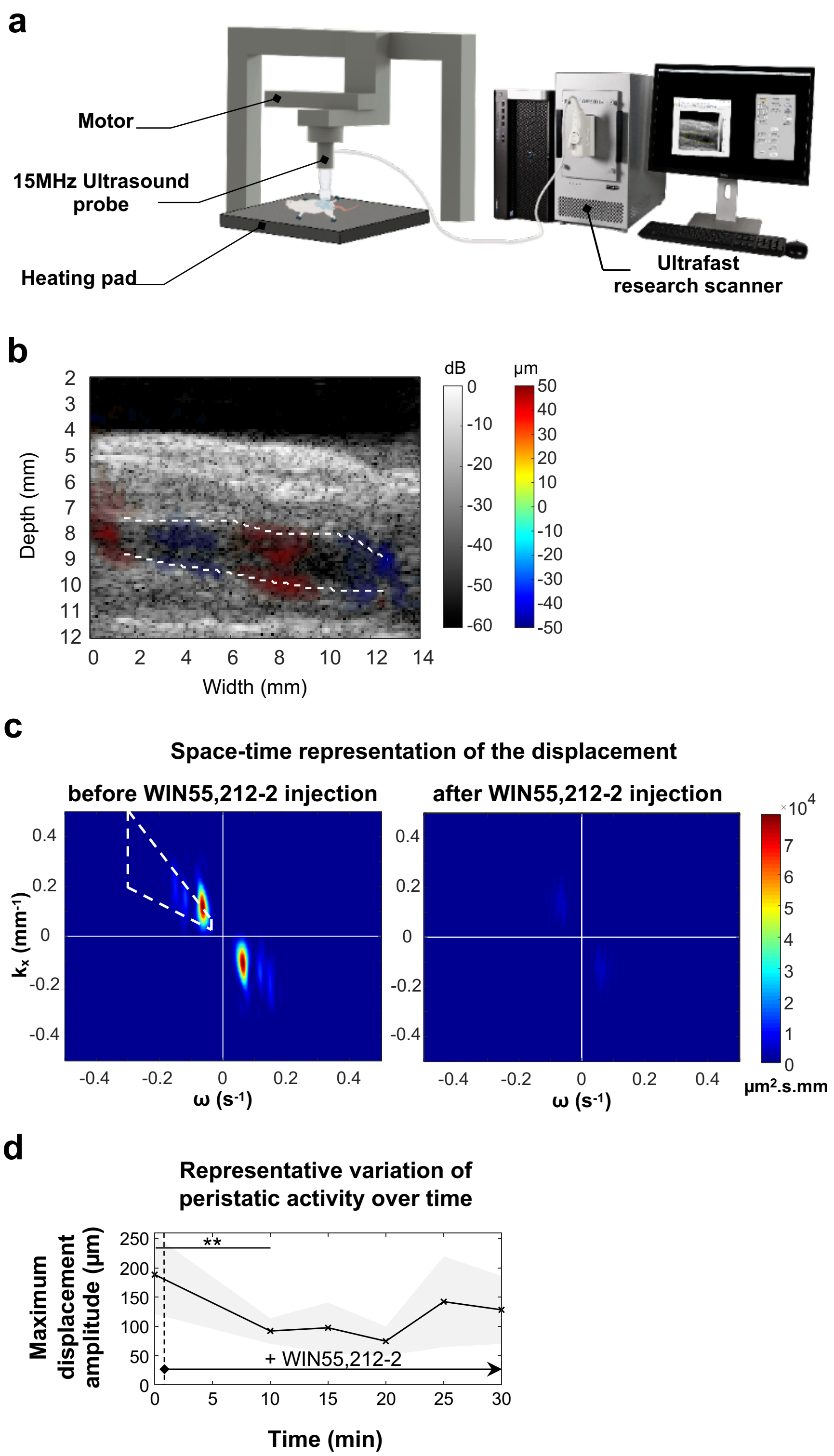

Supplementary Figure 1

Supplementary Figure 1. *In vivo* visualization of endogenous colonic pulsatile waves using ultrasound and inhibition by WIN. a, Scheme of the ultrasonic set-up. **b,** Representative sagittal view of mice colon obtained with B-Mode imaging with sur-imposed color-coded 2D map showing the axial displacement of pulsatile waves. White lines represent colon walls and define the x curvilinear

abscissa used for the space time representation of the displacement in **d**. **c**, k-ω (spatial frequency - temporal frequency) power spectral density (PSD) diagram in WT mice colon before and after injection of WIN associated to the heat maps represented in Fig. 1c. A k-ω diagram is generally used to characterize propagative phenomena: energy at a frequency ω whose wave vector k is different from 0 propagates. **d**, Representative variation of the pulsatile activity over time before and after WIN injection. t0 corresponds to the mean of maximum displacement amplitude of 12 acquisitions before drug injection (n=4, three acquisitions were analyzed for each mouse). T=10, 15, 20, 25 and 30 min corresponds to the mean of maximum displacement amplitude of four acquisitions 10 to 30min after drug injection (n=4, one acquisition was analyzed for each mouse). ** p<0.01 between t0 and t+10 using the Mann-Whitney test.

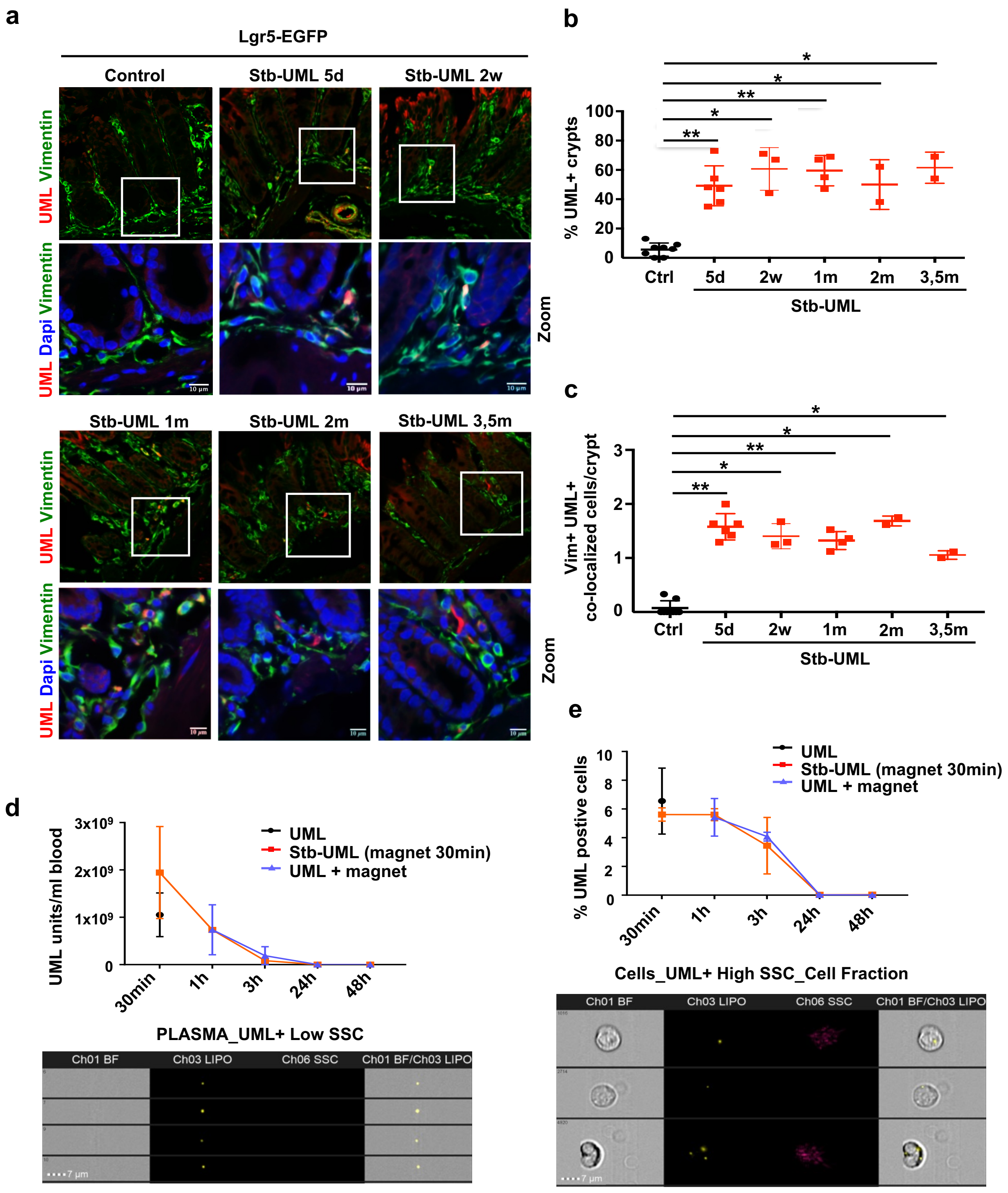

Supplementary Figure 2

Supplementary Figure 2. Stabilization of UML in the colon of mice subjected to pulsed stresses. a, Co-localization of Rhodamine-labelled UML (red) with Vimentin (green) in Lgr5-EGFP mice observed 5 days (n=6 mice), 2 weeks (n=3 mice), 1 month (n=4 mice), 2 months (n=2 mice) and 3,5 months (n=3 mice) after UML injection and subjected to 30 minutes of magnet implantation to stabilize the UML (Stb-UML). Control: mice injected with UML only without magnet implantation (n=8 mice). Small white frames define portions enlarged with a scale bar of 10µm. **b**, Quantification of **a.** Percentage of total UML positive crypts. Mann-Whitney test; * p<0.05, ** p<0.01. **c,** Quantification of UML and Vimentin co-localization**.** Percentage of UML and Vimentin positive cells per crypt. Mann-Whitney

test; * p<0.05, ** p<0.01. **d,** Quantification of UML units per milliliter of blood in the plasma of mice injected with UML without magnet implantation (UML), injected with UML that are stabilized in the colon after implantation of a magnet for 30 minutes (Stb-UML) and injected with UML plus permanent magnet implantation (UML + magnet), n=3 mice/condition. **e,** Percentage of blood cells that have internalized the UML in mice injected with UML without magnet implantation (UML), injected with UML that are stabilized in the colon after implantation of a magnet for 30 minutes (Stb-UML) and injected with UML plus permanent magnet implantation (UML + magnet), n=3 mice/condition. Statistical significance determined using the Holm-Sidak method. Error bars: standard deviation.

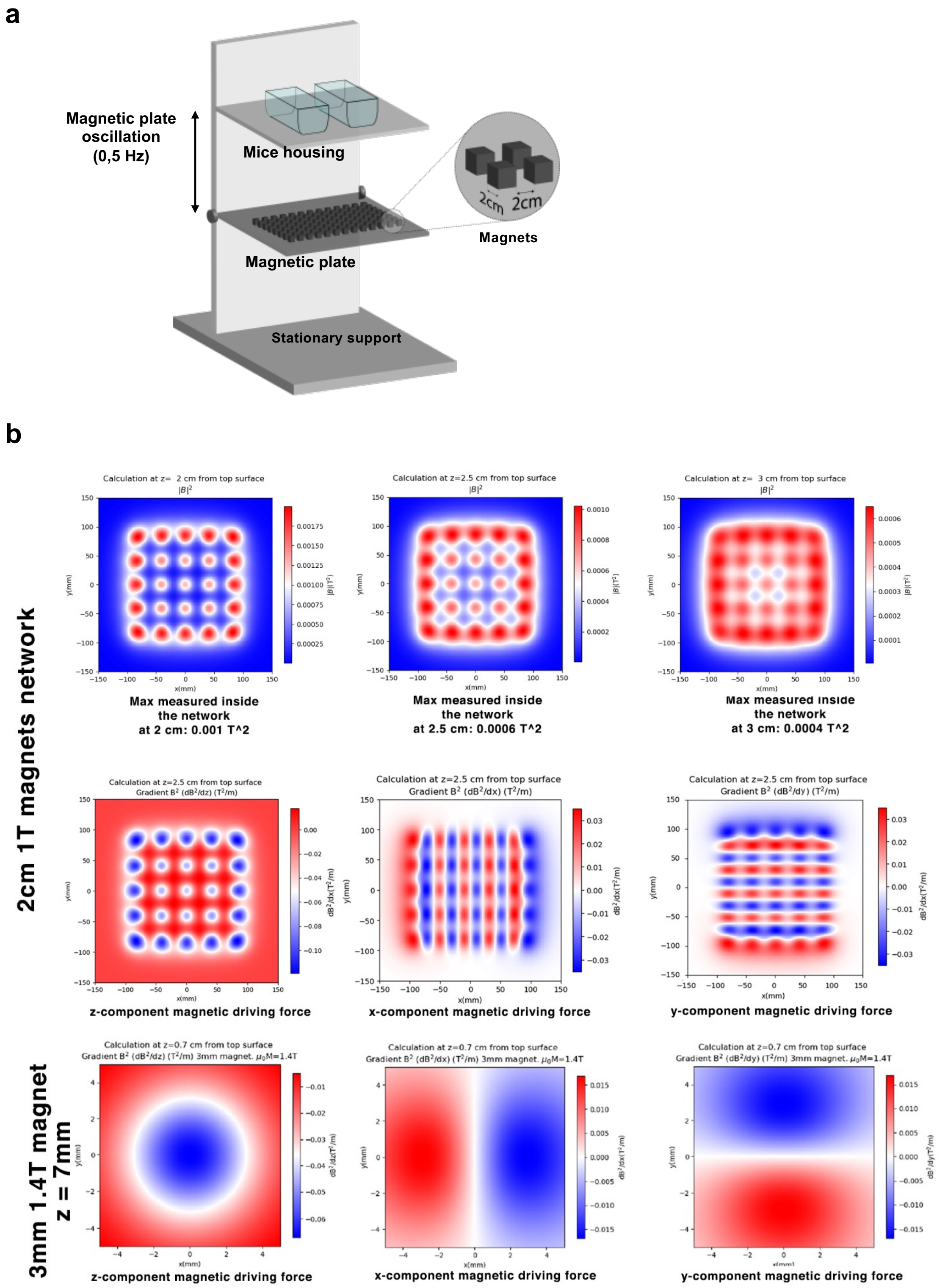

Supplementary Figure 3

Supplementary Figure 3. Custom-built system and simulations for the application of pulsated magnetic deformation. a, Scheme of the magnetic field gradient set-up. **b**, Magnetic fields produced by a 2D network of 2cm 1T magnets separated by 2cm and by the 3mm 1.4T magnet at 7mm of distance. **Up**, Predicted and measured magnetic fields at different distances z from the magnet network plane top surface. **Middle**, B^2 gradient driving force of the network along z (perpendicular to the network plan dimensions), and along x and y (parallel to the network plan dimensions), at z=

2.5cm distance from the 2D magnet network plan upper surface. **Down,** B^2 gradient driving force of the 3mm 1.4T magnet along z (parallel to the magnetic dipole), and along x and y (perpendicular to the magnetic dipole), at z= 7mm distance from its upper surface.

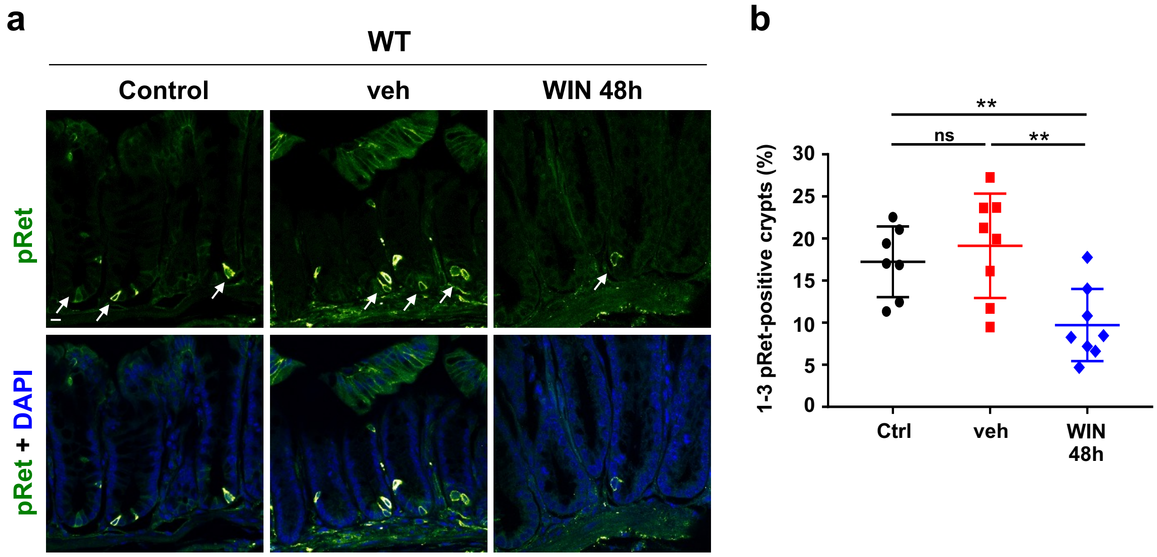

Supplementary Figure 4

Supplementary Figure 4. The cannabinoid agonist WIN decreases the physiological signal of pRet in the colon. a, Effect of the pulsatile inhibitor cannabinoïde WIN on the physiological signal of pY1062 Ret kinase (percentage of crypts with 1 to 3 pRet positive cells) in WT mice after treatment for 48 hours. Control non-treated mice (n=7 mice); veh: treated with the vehicle of WIN (n=8 mice); WIN 48h: mice treated with WIN for 48h (n=8 mice). White arrows show 1-3 pRet+ cryps. Scale bar is 10µm. **b,** Quantification of **a**. Values represent the percentage of colon crypts that display 1 to 3 pRet kinase positive cells per crypt. Mann-Whitney test; ** p<0.01. Error bars: standard deviation.

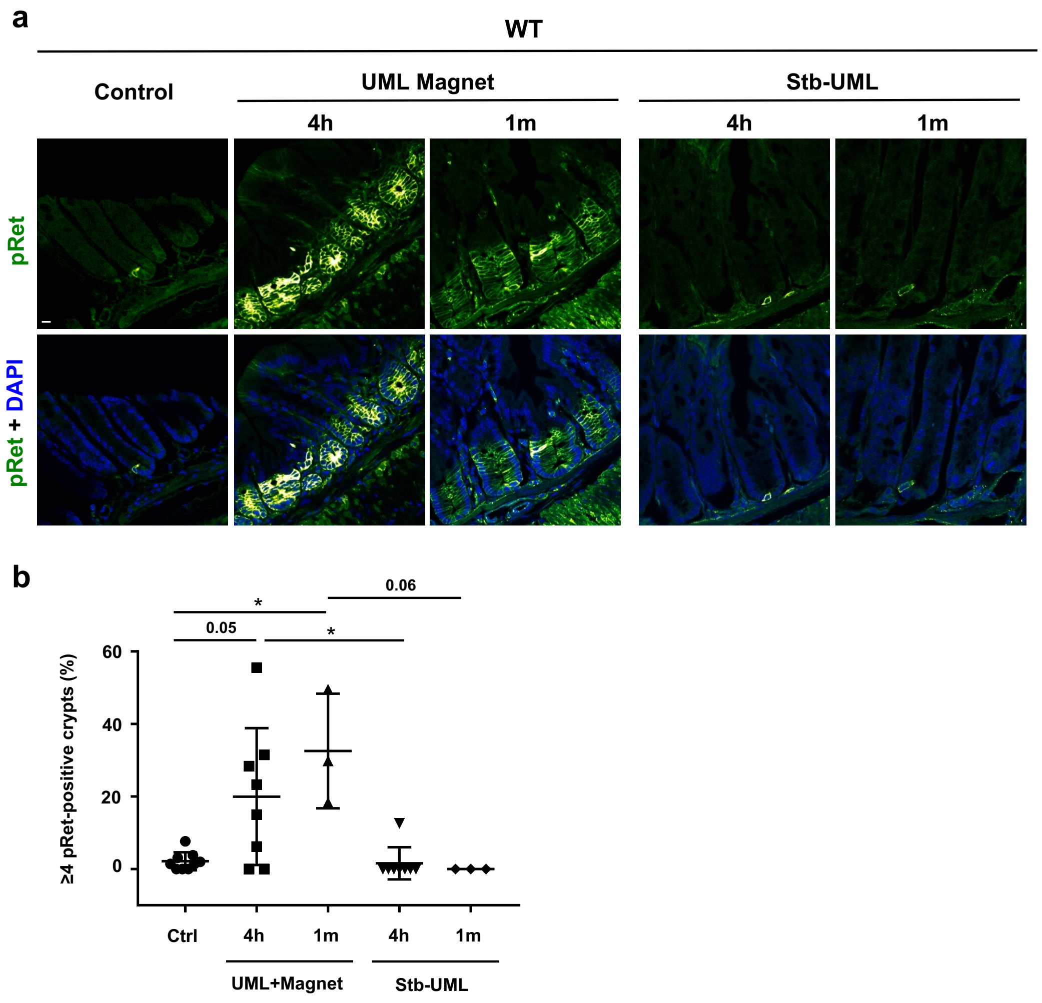

Supplementary Figure 5

Supplementary Figure 5. Colon magnetization after 30 minutes of magnet implantation does not induce pathological activation of the Ret signaling pathway. a, Representative immunostaining images of the physiological (1-3 pRet+ cells per crypt) and the pathological (≥4 pRet+ cells per crypt) pY1062 Ret kinase signal in green in WT mice. UML Magnet: mice injected with UML plus permanent magnet implantation for 4h or 1 month. Stb-UML: mice injected with UML plus magnet implantation for only 30 minutes after UML injection and sacrifice 4h or 1 month later. **b,** Quantification of the colon crypts that display ≥4 pRet+ cells per crypt in **a**. Mann-Whitney test; * $p<0.05$. Error bars: standard deviation.

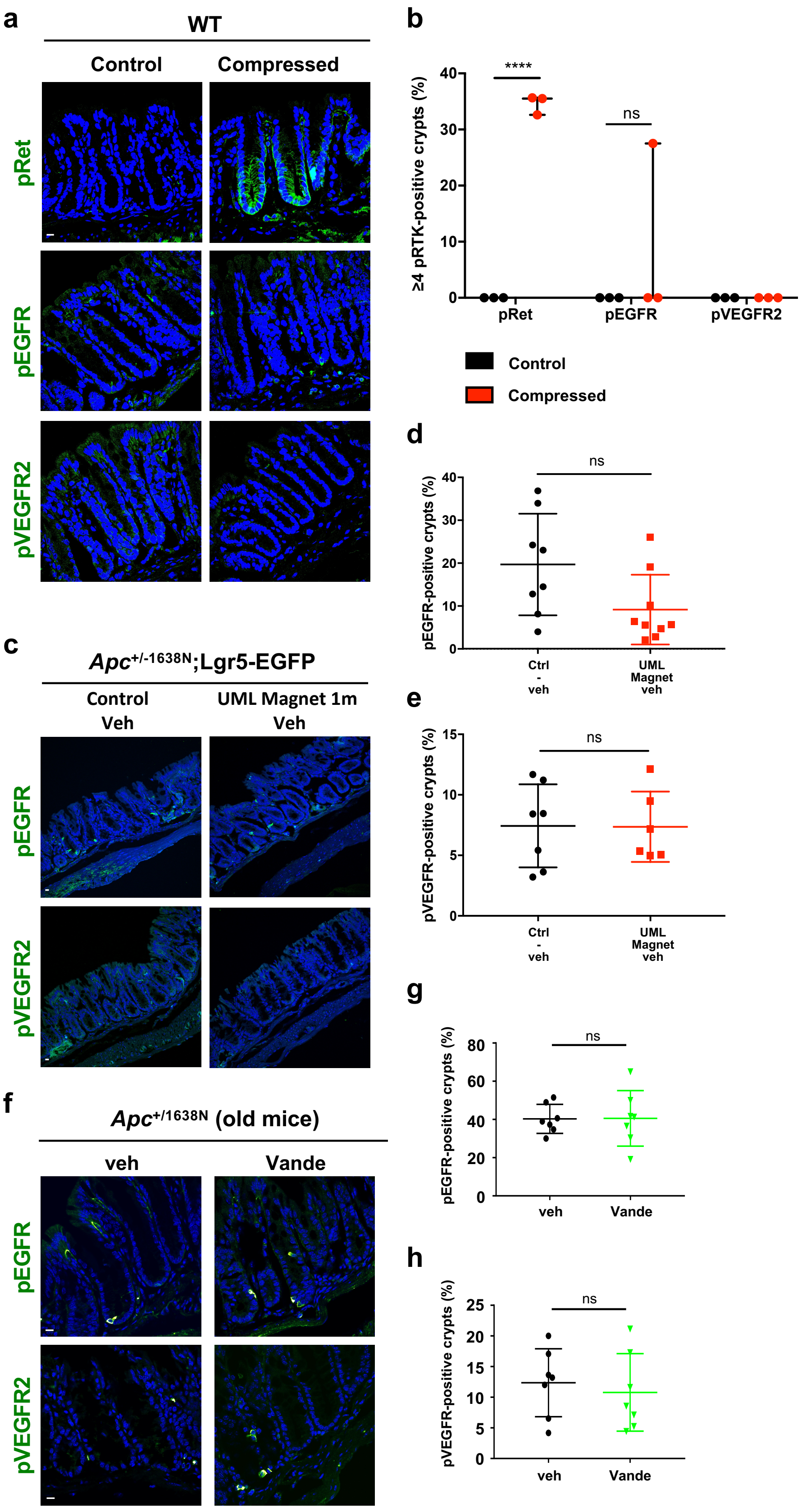

Supplementary Figure 6

Supplementary Figure 6. Mechanical pressure activation of tyrosine kinase receptors (RTKs) targets of Vande is Ret specific. a, RTKs phosphorylation observed after 1 min of 1kPa *ex vivo* mechanical compression on WT mice colon explants. Scale bar is 10µm. **b,** Quantification of RTKs phosphorylation

after 1 min of 1 kPa compression, n=3 mice for each condition. Statistical significance determined using the Holm-Sidak method, with alpha = 0.05. Adjusted p (pRet, Ctrl vs Compressed) < 0.0001. **c,** pEGFR and pVEGFR2 phosphorylation observed after 1 month of *in vivo* permanent mechanical pressure on Apc;Lgr5-EGFP mice. Scale bar is 10µm. **d,** Quantitative analysis of pEGFR signal. Percentage of pEGFR+ crypts per mouse. Mann-Whitney test. **e,** Quantitative analysis of pVEGFR2 signal. Percentage of pVEGFR2+ crypts per mouse. Mann-Whitney test. **f,** pEGFR and pVEGFR2 phosphorylation observed in Apc and Apc;N1Cre-ERT2 old mice (16 month-old). pEGFR and pVEGFR2 antibody staining in colon crypts of mice treated with vehicle (n=6 mice) and Vande (n=7 mice). Scale bar is 10µm. **g,** Quantification analysis of pEGFR. Percentage of pEGFR+ crypts per mouse. **(h)** Quantification analysis of pVEGFR2. Percentage of pVEGFR2+ crypts per mouse. Mann-Whitney test. Error bars: standard deviation.

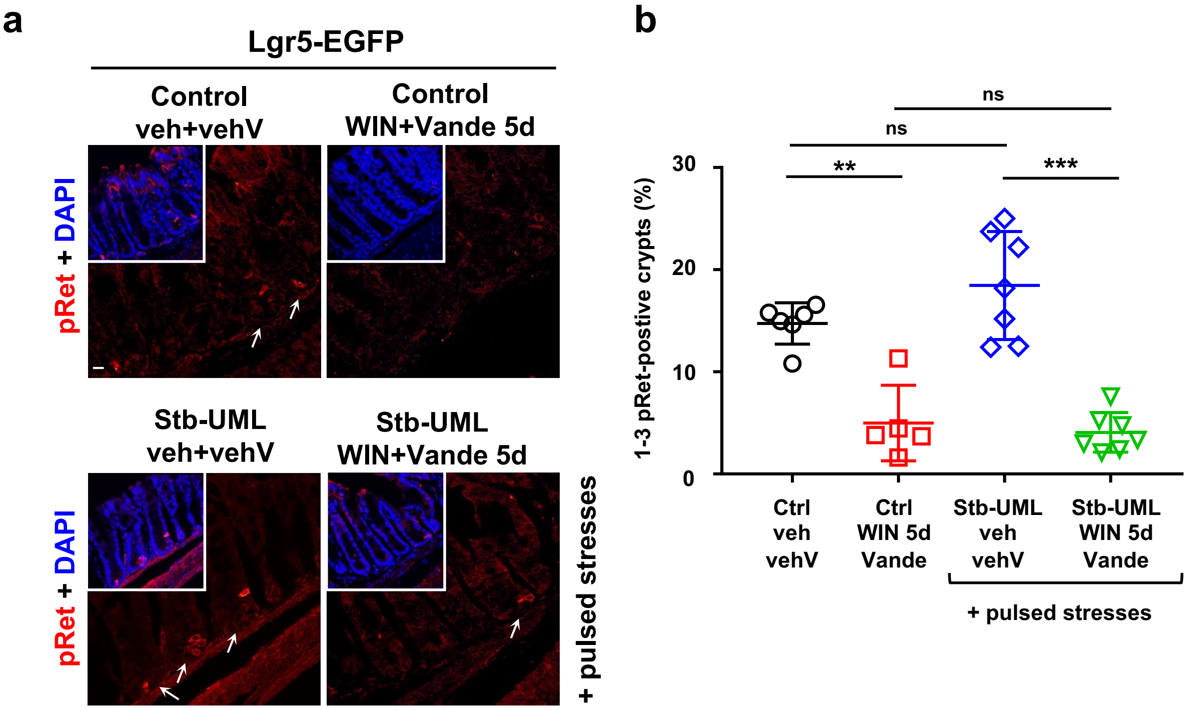

Supplementary Figure 7

Supplementary Figure 7. Treatment with the cannabinoid agonist WIN and the inhibitor Vande decreases the activation of pRet kinase. a, Levels of Y1062 Ret kinase phosphorylation after treatment with both the cannabinoid agonist WIN and the Ret inhibitor Vande for 5 days in the Lgr5-EGFP mouse model. Ctrl: mice injected with UML only without magnet implantation, Stb-UML: mice injected with UML and subjected to 30 minutes of magnet implantation to stabilize the UML, veh: injected with the vehicle of WIN, WIN: injected with WIN, vehV: implemented with the vehicle of Vande, Vande: treated with Vande. White arrows show 1-3 pRet+ crypts. Scale bar is 10μm. **b**, Quantification of **b**. Percentage of crypts that show 1-3 pRet positive cells. Mann-Whitney test; ** p<0.01; *** p<0.001; ns: not significant. Error bars: standard deviation.

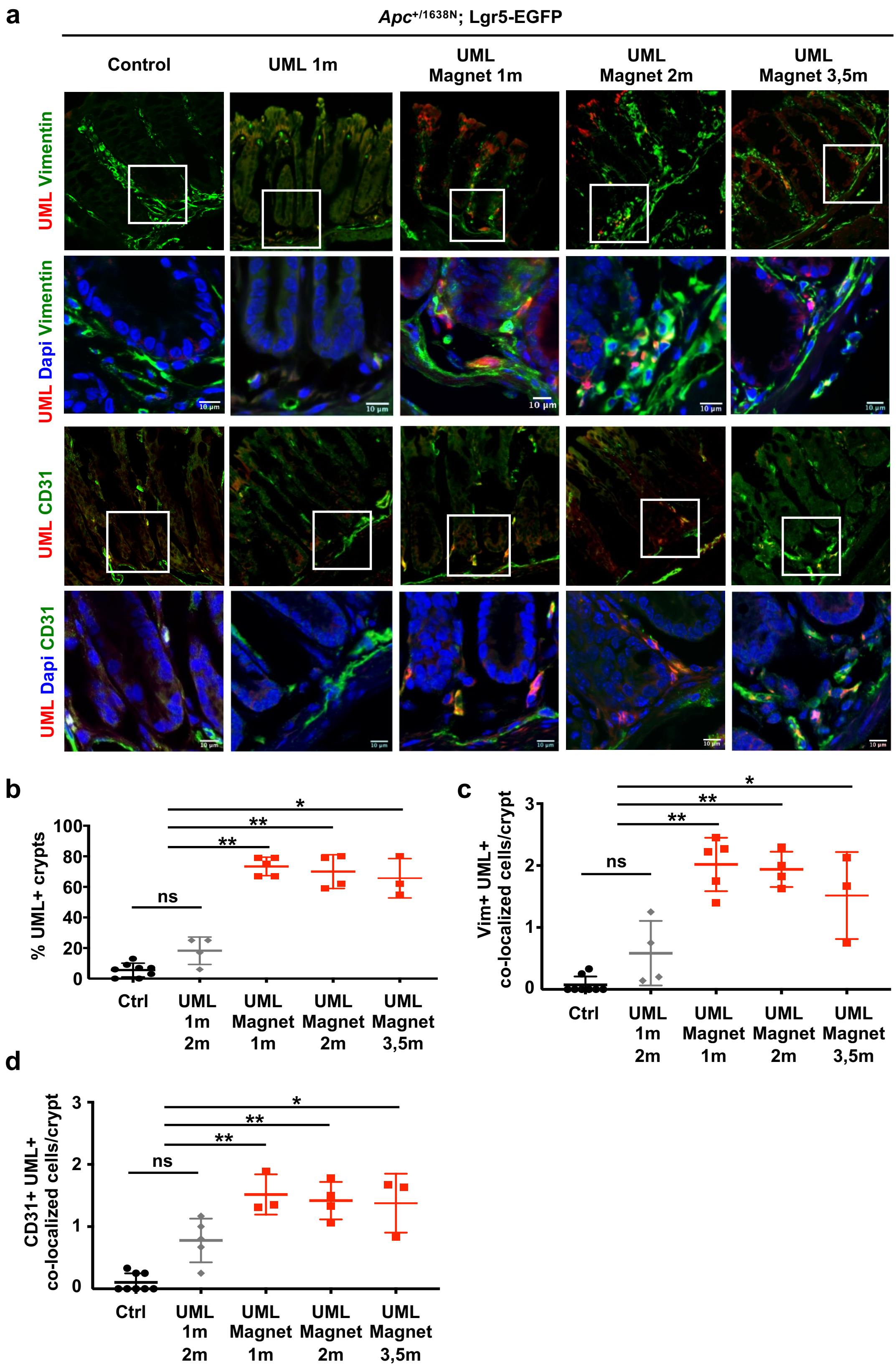

Supplementary Figure 8

Supplementary Figure 8. Stabilization of UML in the colon after injection of mice subjected to a permanent stress. a, Co-localization of Rhodamine-labelled UML (red) with Vimentin and CD31 (green) in Apc;Lgr5-EGFP mice after application of a permanent magnetic compression mimicking

tumor growth pressure for 1, 2 and 3,5 months. Control: mice without UML injection (n=8 mice); UML 1m: mice injected with UML without magnet implantation and analyzed 1 month later (n=4 mice); UML Magnet: mice injected with UML plus permanent magnet implantation for 1 month (n=5 mice), 2 months (n=4 mice) and 3,5 months (n=3 mice). Small white frames define portions enlarged with a scale bar of 10μm. **b**, Quantification of **a.** Percentage of total UML positive crypts. Mann-Whitney test; * $p<0.05$, ** $p<0.01$. **c**, Quantification of UML and Vimentin co-localization**.** Percentage of UML and Vimentin positive cells per crypt. Mann-Whitney test; * $p<0.05$, ** $p<0.01$. **d**, Quantification of UML and CD31 co-localization**.** Percentage of UML and CD31 positive cells per crypt. Mann-Whitney test; * $p<0.05$, ** $p<0.01$. Error bars: standard deviation.

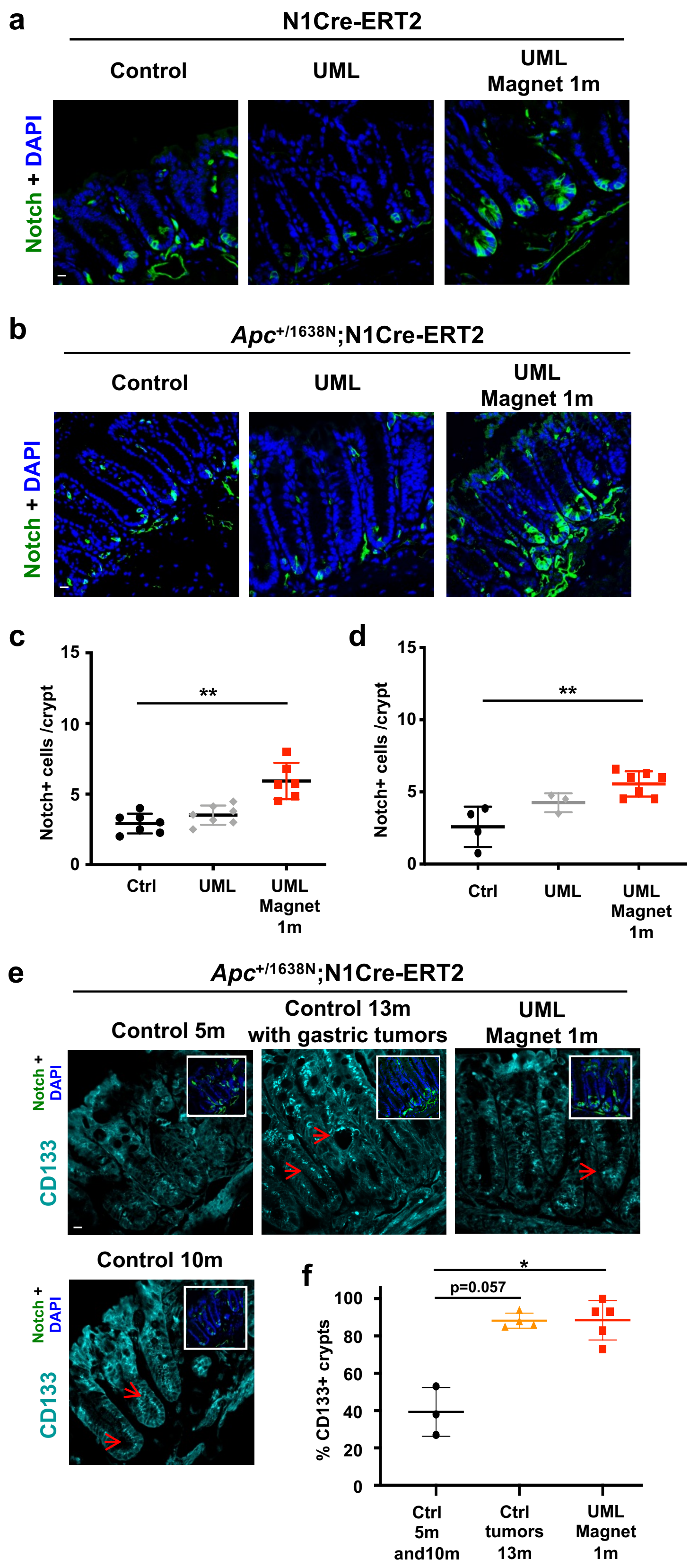

Supplementary Figure 9

Supplementary Figure 9. Permanent tumorous mechanical stress over-stimulates Notch1 PC and CD133 CSC production, *in vivo*. a, Mechanical stimulation of Notch1 PC in N1Cre-ERT2 mice following

permanent mechanically colon stress pressure, *in vivo*. Control: mice without UML injection (n=7 mice); UML: mice injected with UML without magnet implantation (n=7 mice); UML Magnet: mice injected with UML plus permanent magnet implantation for 1 month (n=6 mice). Scale bar is 10μm. **b**, Mechanical stimulation of Notch1 PC in Apc;N1Cre-ERT2 mice following permanent mechanically colon stress pressure, *in vivo*. Control: mice without UML injection (n=4 mice); UML: mice injected with UML without magnet implantation (n=3 mice); UML Magnet: mice injected with UML plus permanent magnet implantation for 1 month (n=7 mice). Scale bar is 10μm. **c**, Quantification of **a**. Mean number of Notch1-EGFP+ cells per crypt and per mouse. Mann-Whitney test: **p<0.01. **d**, Quantification of **b**. Mean number of Notch1-EGFP+ cells per crypt and per mouse. Mann-Whitney test: **p<0.01. **e**, Mechanical stimulation of CD133+ CSC multiplication in Apc;N1Cre-ERT2 mice. CD133 antibody staining (red arrows) in control 5 months and 10 months (n=3 mice), in 13 month-old mice having gastric tumors (small intestine and colon) (n=4 mouse), and in mice injected with UML with magnetic pressure for 1 month (n=5 mice). Small white frames show similar images with Notch1-EGFP and nuclei staining. Scale bar is 10μm. **f**, Quantification analysis of **e**. Values represent the percentage of CD133+ crypts per mouse. Mann-Whitney test: *p< 0.05. Error bars: standard deviation.

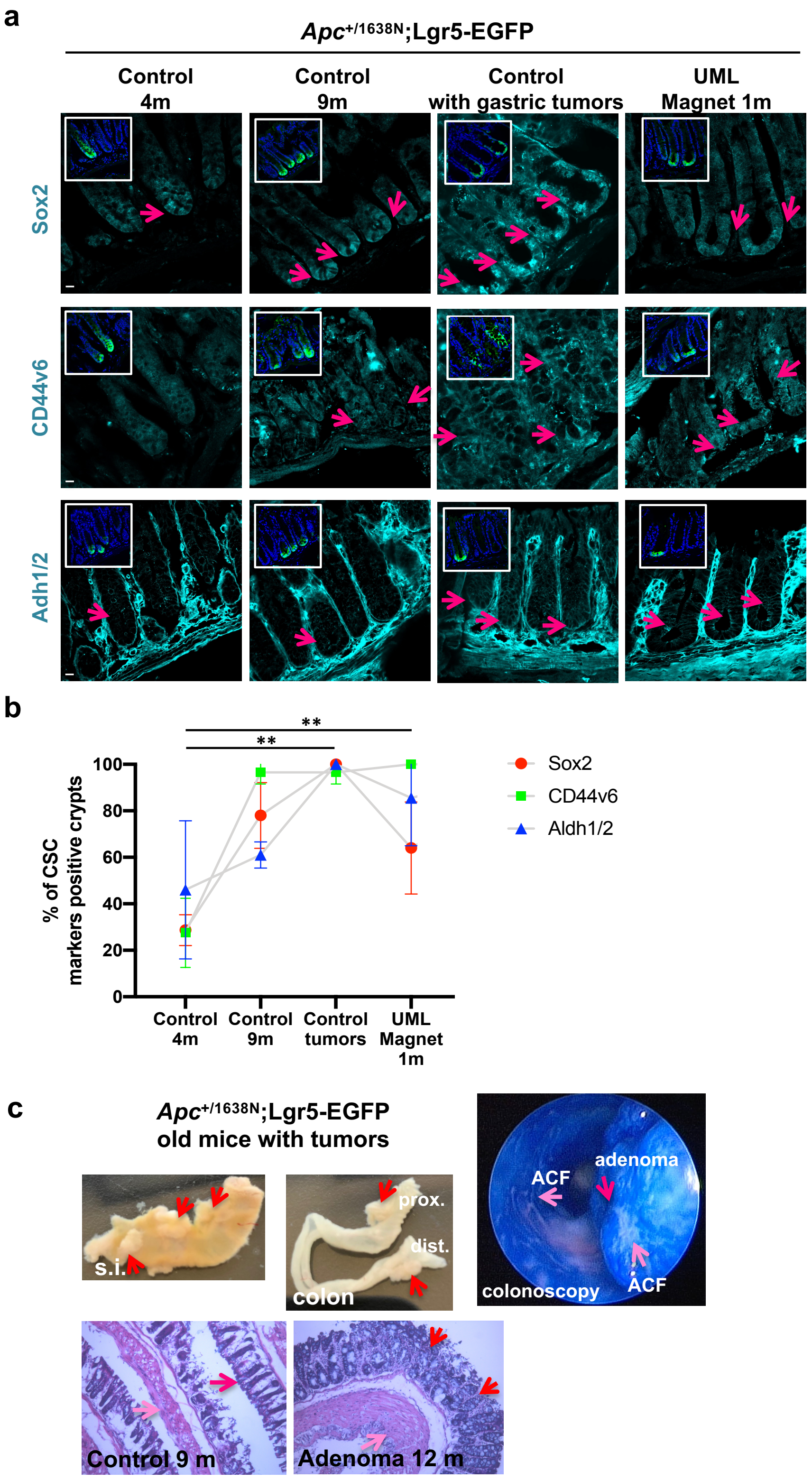

Supplementary Figure 10

Supplementary Figure 10. Increased expression of Sox2, CD44v6 and Aldh1/2 CSC markers in Apc;Lgr5-EGFP mice after permanent mechanical magnetic pressure *in vivo*. a, Apc;Lgr5-EGFP mice were analysed for Sox2, CD44v6 and Aldh1/2 CSC markers expression, in control 4 months- and 9 month-old mice control with gastric tumors and after permanent *in vivo* mechanical magnetic pressure for 1 month. Positive crypts staining are visualised by red arrows. Small white frames show

similar images with Lgr5-EGFP and nuclei staining. Scale bar is 10μm. **b**, Compilation graph of the percentage of CSC marker positive crypts in control, control with tumors and under magnetic pressure. Sox2 values are in red, CD44v6 in green and Aldh1/2 in blue. Mann-Whitney test for comparison of the mean percentage of each CSC marker-positive crypts between 4 month-old control mice and i) control mice with gastric tumors (**$p<0.01$), and ii) UML Magnet for 1month, (**$p<0.01$). **c**, Organs from a 12 month-old Apc;Lgr5-EGFP mice: small intestine (s.i.) with multiple tumors (3 red arrows); colon with 2 tumors localised in the proximal and terminal regions (2 red arrows). Colonoscopy: identification of a large adenoma in the colon (red arrow) and ACF visible on the colon wall and on the adenoma (2 pink arrows); histologic characterization using haematoxylin/eosine staining of the colon of the 12-months mice with tumors, including the large adenoma characterized by aberrant crypts (2 red arrows); and a thick mucosa (1 pink arrow); compared to the 9 month-old mice with no adenoma, a thin mucosa (1 pink arrow) and normal crypts (1 red arrow). Error bars: standard deviation.

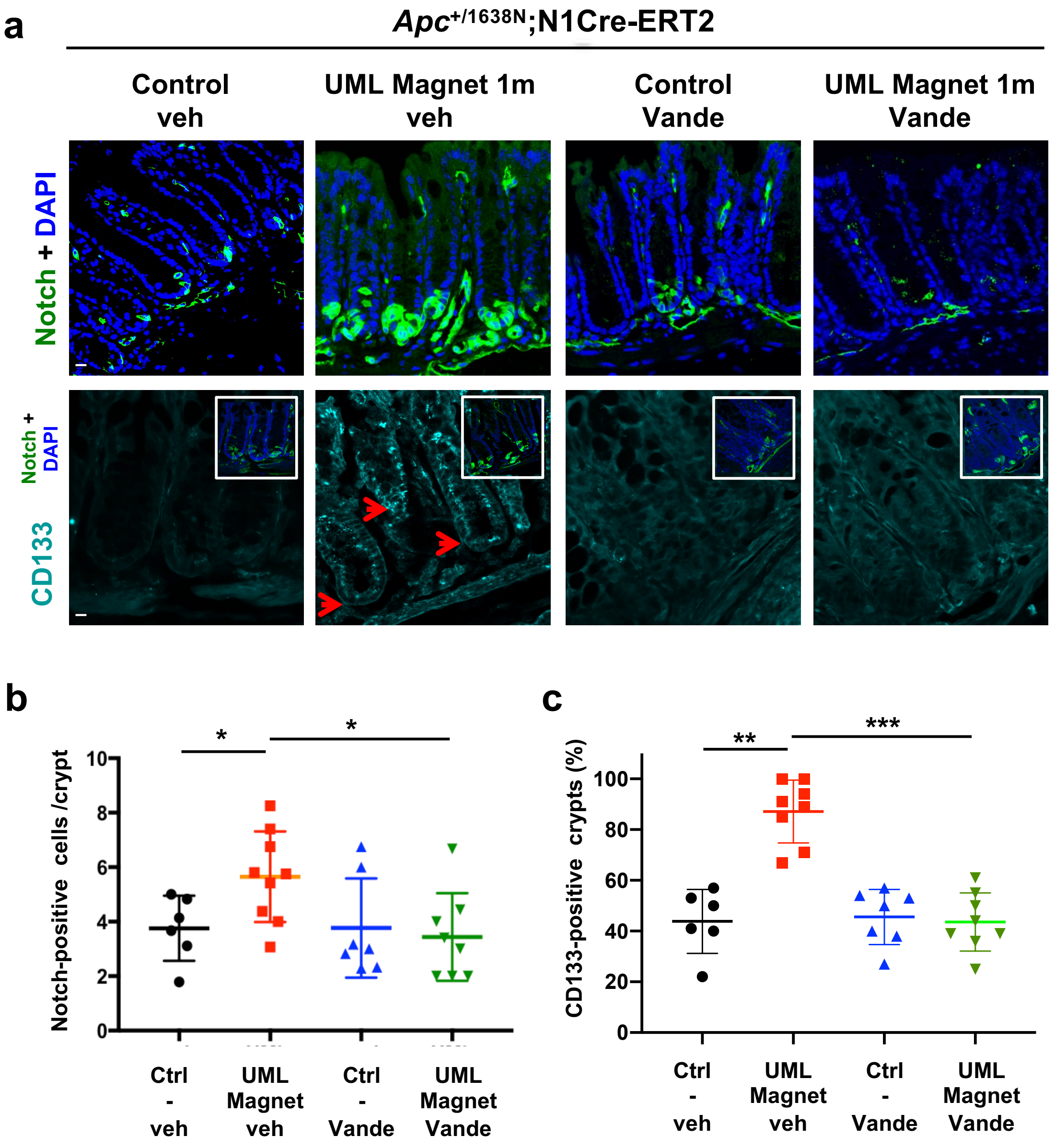

Supplementary Figure 11

Supplementary Figure 11. The mechanical stimulation of Notch1-GFP PC and CSC is pRet dependent in Apc;N1Cre-ERT2 mice. a, Mechanical stimulation of Notch1-GFP positive PC and CD133 CSC in Apc;N1Cre-ERT2 mice. Control Veh: mice without UML injection treated for 1 month with vehicle (n=6 mice); UML Magnet 1m Veh: mice injected with UML plus permanent magnet implantation and treated with vehicle for 1 month (n=9 mice); Control Vande: mice without UML injection treated for 1 month with Vande (n=7 mice); UML Magnet 1m Vande: mice injected with UML plus permanent magnet implantation and treated with Vande for 1 month (n=8 mice). N=2 experiments. CD133+ stained cells: red arrows. White box show similar images with Notch1-EGFP and nuclei staining. Scale bar is 10µm. **b**, Quantitative analysis of (**a** up). Mean number of Notch1-EGFP positive PC per crypt and per mouse. Mann-Whitney test; *p<0.05. **c**, Quantitative analysis of (**a** down). Values represent the percentage of CD133+ cells per mouse. Mann-Whitney test; **p<0.01; ***p<0.001. Error bars: standard deviation.

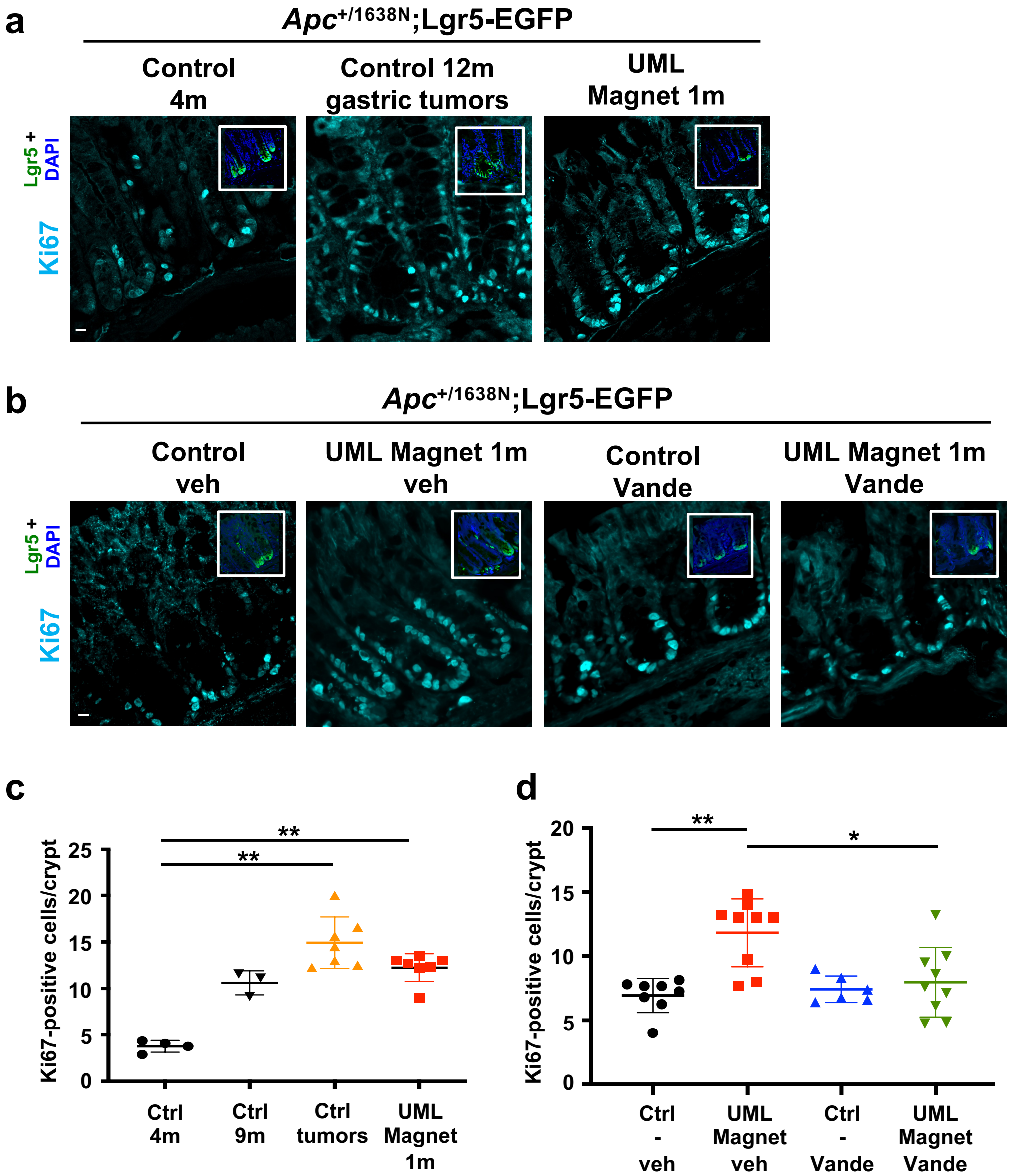

Supplementary Figure 12

Supplementary Figure 12. Inhibition of the mechanical stimulation of the Ki67 proliferative marker in Apc;Lgr5-EGFP mice by Ret inhibitor Vande. a, Ki67 antibody staining of 4 month-old Apc;Lgr5-EGFP control mice (n=6 mice), 9 month-old control mice (n=3 mice), 12/13 month-old control mice with gastric tumors (n=7 mice), and mice injected with UML under magnetic pressure for 1 month (n=7 mice). Ki67+ cells are shown in cyan; white small boxes include the similar images showing Lgr5-EGFP and nuclei staining in blue. Scale bar is 10µm. **b**, Ki67 antibody staining of 4 month-old Apc;Lgr5-EGFP control mice treated for 1 month with vehicle (n=8 mice), vehicle and injected with UML+magnet for 1 month, (n=9 mice), vehicle+ Vande (n=6 mice), and vehicle+Vande and injected with UML+magnet for 1 month (n=9 mice). **c**, Quantitative analysis by Mann-Whitney test two-tailed of **a**, for comparison of the mean number of Ki67+ cells per crypt between 4 month-old control mice with control mice with gastric tumors (**$p< 0.01$), and with UML Magnet for 1month (**$p< 0.01$). **d**, Quantitative analysis and Mann-Whitney test two-tailed of **b**, for UML magnet/vehicle mice compared to control vehicle (**$p<0.01$) and to UML magnet/Vande treated mice (*$p<0.05$). Error bars: standard deviation.

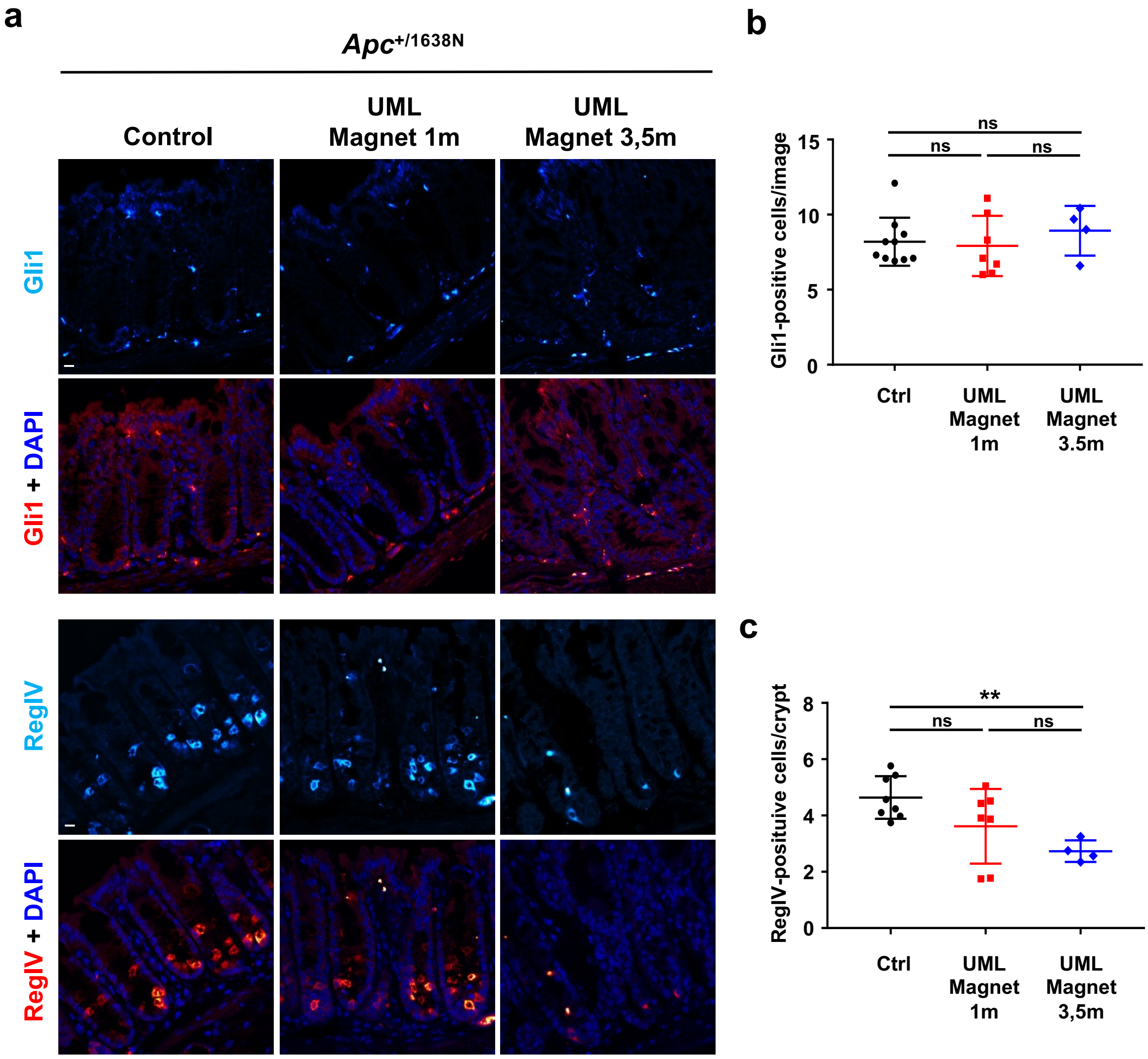

Supplementary Figure 13

Supplementary Figure 13. A permanent mechanical stimulation of the colon epithelia has opposite effects on the different components of the cellular niche in Apc-deficient mice. a, Top, levels of Gli1 positive mesenchymal niche cells per image observed by immunofluorescence after mechanical compression of the colon tissue for 1 month (n=7 mice) and 3.5 months (n=4 mice), compared to control (n=10 mice,). Down, levels of RegIV positive niche cells per crypt observed by immunofluorescence after mechanical compression of the colon tissue for 1 month (n=7 mice) and 3.5 months (n=4 mice) compared to control (n=8 mice). Scale bar is 10µm. **b,** Quantification of **a** (up). Mean number of Gli1+ cells per image per mouse (n=10 images per mouse analysed). Mann-Whitney test; ns=not significant. **c**, Quantification of **a** (down). Mean number of RegIV+ cells per crypt. Mann-Whitney test; ** p<0.01. Error bars: standard deviation.

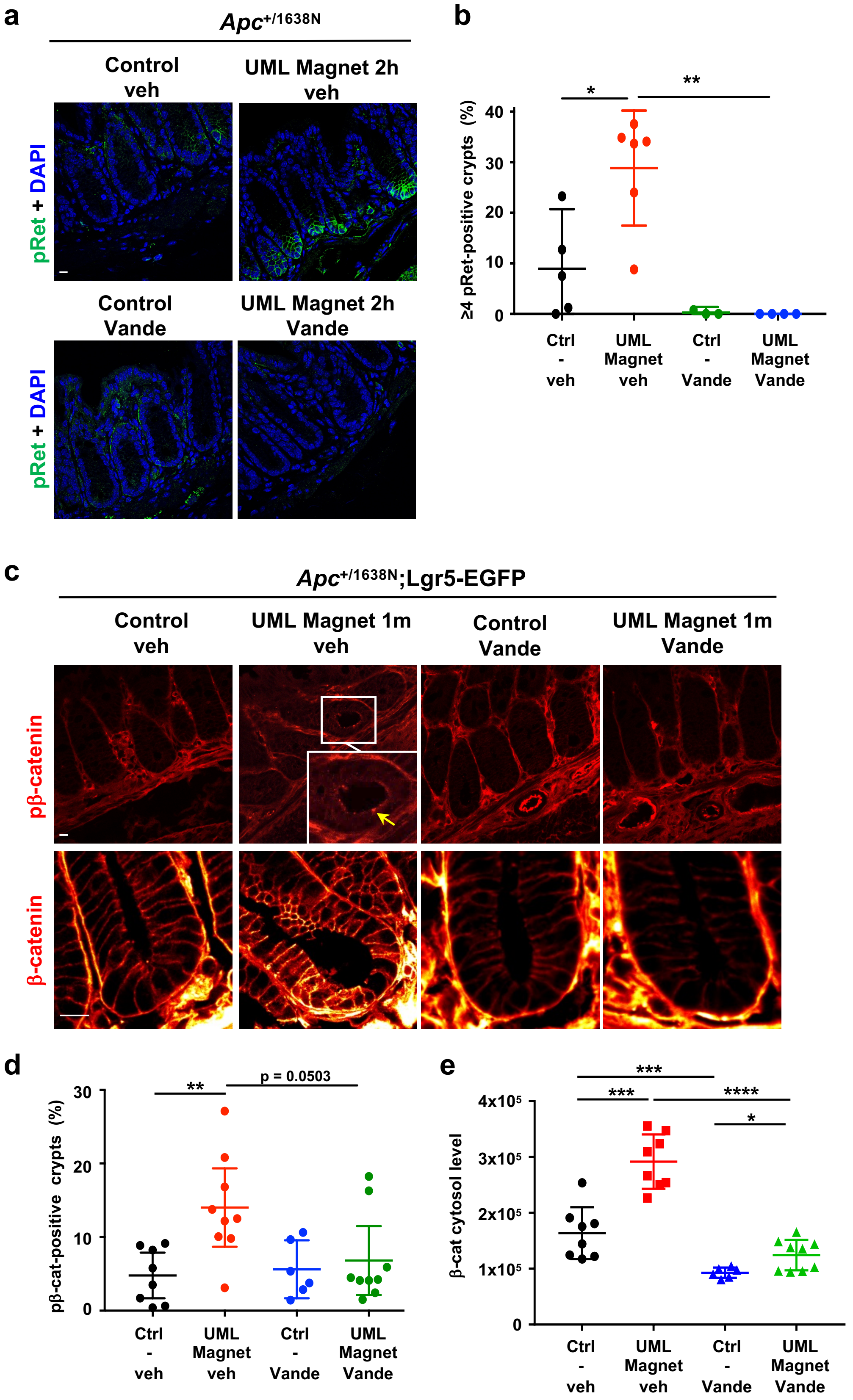

Supplementary Figure 14

Supplementary Figure 14. Pharmacological inhibition of the Ret/β-cat pathway with the pRet inhibitor Vande in Apc mice. a, Ret phosphorylation after 2 hours of permanent mechanical pressure

with or without Vande treatment. Scale bar is 10µm. **b**, Quantification of Ret phosphorylation after 2 hours of permanent mechanical pressure with or without Vande treatment, n=3-6 mice for each condition. Mann-Whitney test: * p< 0.05 and ** p<0.01. **c**, β-cat phosphorylation and cytosolic accumulation in Apc;Lgr5-EGFP mice colon after 1 month *in vivo* permanent mechanical pressure with or without Vande treatment, n=6-9 mice for each condition. Scale bars are 10µm. **d**, Quantitative analysis of the pY654 β-cat signal. Percentage of pY654 β-cat positive crypts per mouse. Mann-Whitney test; **p<0.01. **e**, Quantitative analysis of the β-cat signal. The values represent the Mean Integrated Density (IntDen) signal of a minimum of 10 crypts per mouse measured with ImageJ. Mann-Whitney test; *p<0.05, ***p<0.001 and ****p<0.0001. Error bars: standard deviation.

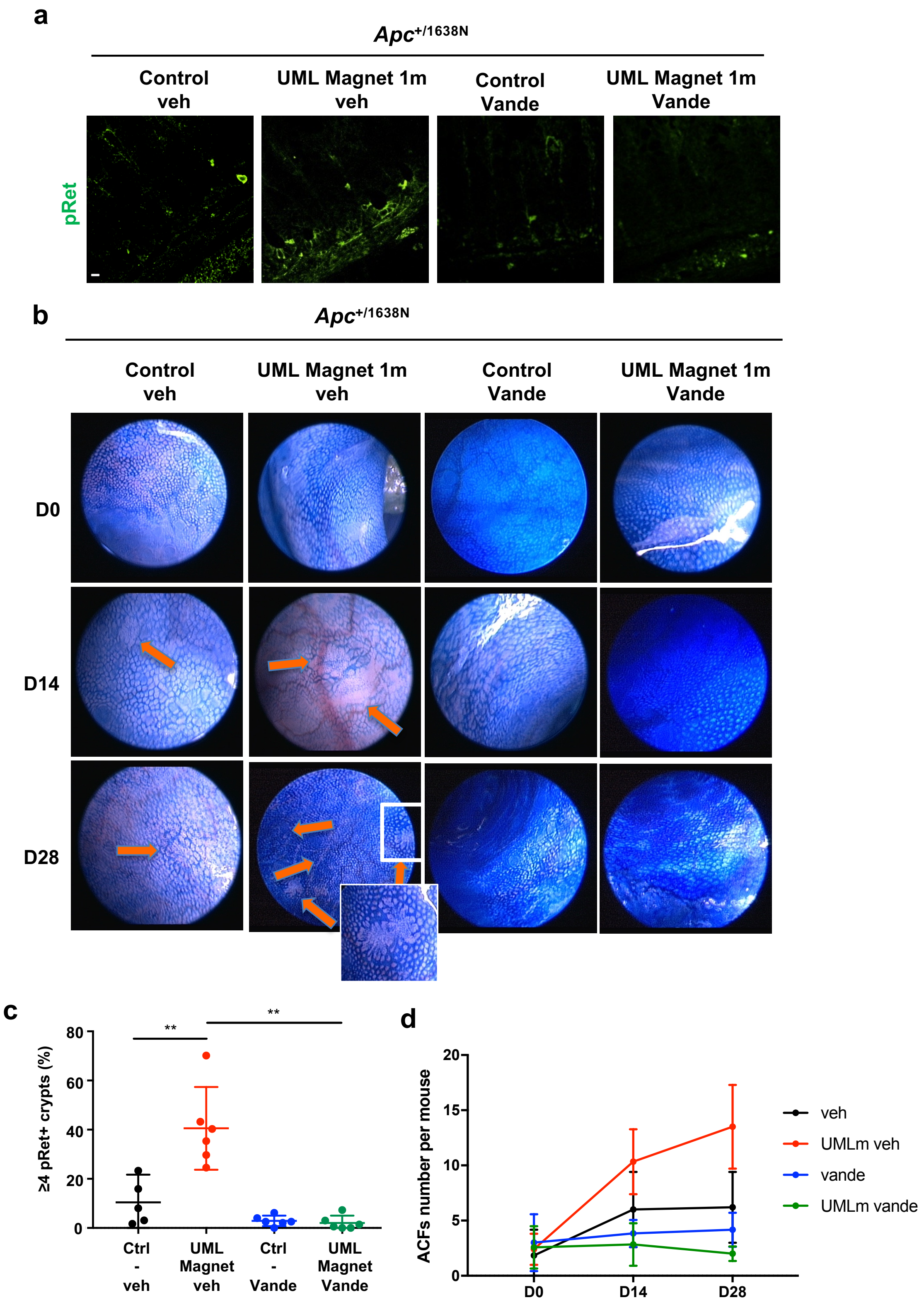

Supplementary Figure 15

Supplementary Figure 15. The pharmacological inhibition of the Ret/β-cat pathway with Vande inhibits the mechanical stimulation of ACFs in Apc mice. a, Levels of Ret phosphorylation (in green) after 1 month of *in vivo* permanent mechanical pressure with or without Vande treatment in the Apc

mice. Scale bar is 10µm. **b**, Live imaging of ACF (orange arrows) at different times of the experiment in Apc mice subjected to 1 month of permanent mechanical pressure with or without Vande treatment. **c**, Quantitative counting of ≥4 pRet+ cells per crypt signal after 1 month of *in vivo* permanent mechanical pressure in presence or absence of Vande gavage, n=5-6 mice for each condition. N=2 experiments. Mann-Whitney test; **p<0.01. **d**, ACF number counting at different times until 1 month of permanent pressure on the colon of Apc mice with or without Vande treatment, n=6-7 mice/condition. N=2 experiments. Statistical significance determined using the Holm-Sidak method, with alpha = 0.05. Adjusted p (D28, UMLm veh vs. veh) < 0.05, adjusted p (D14, UMLm vande vs. UMLm veh) < 0.001, adjusted p (D28, UMLm vande vs. UMLm veh) < 0.0001. Error bars: standard deviation, except for d in which it is standard error to the mean.

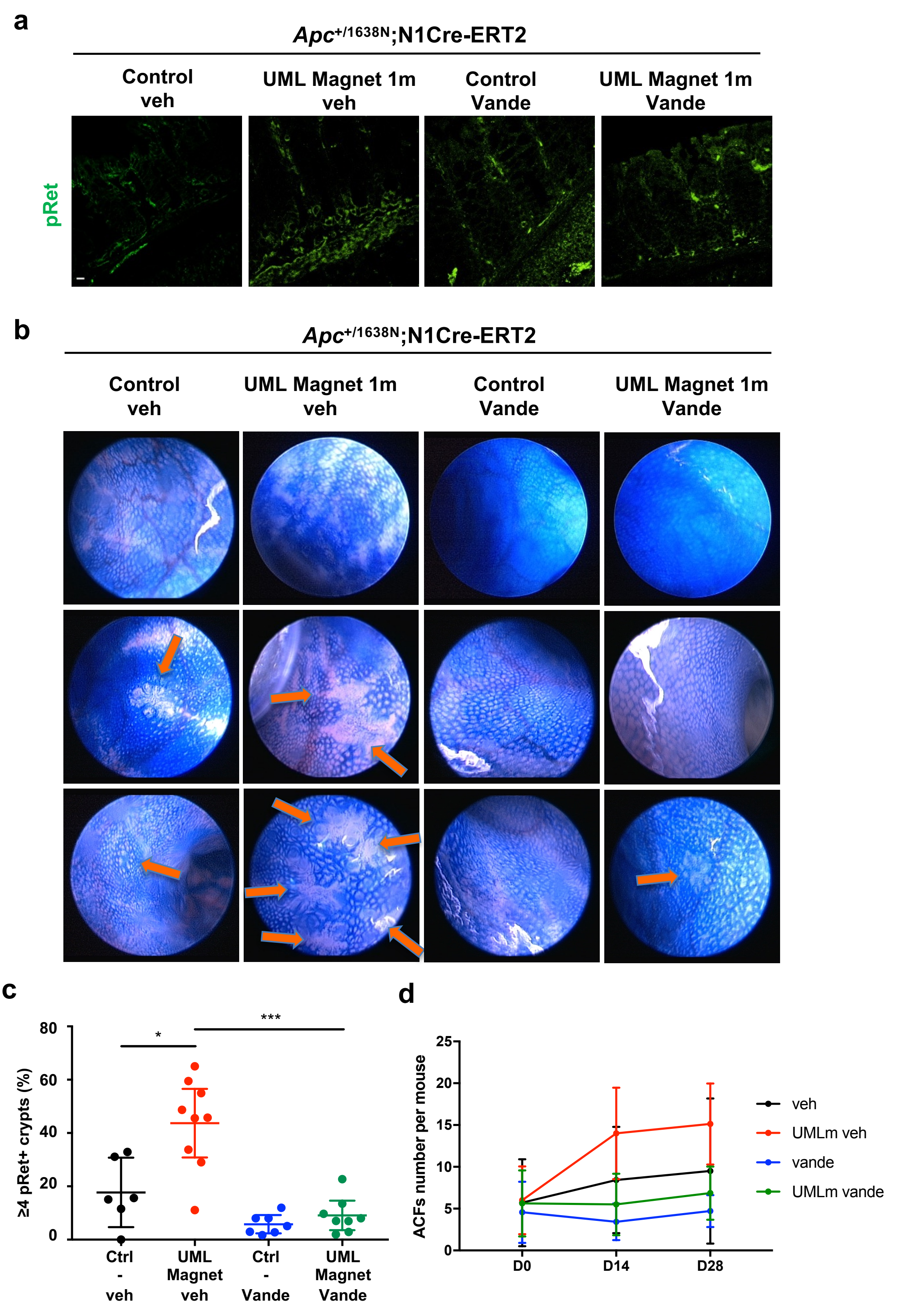

Supplementary Figure 16

Supplementary Figure 16. The pharmacological inhibition of the Ret/β-cat pathway with Vande inhibits the mechanical stimulation of ACFs in Apc$^{+/1638N}$;N1Cre-ERT2 mice. a, Levels of Ret phosphorylation (in green) after 1 month of *in vivo* permanent mechanical pressure with or without

Vande treatment in the Apc;N1Cre-ERT2 mice. Scale bar is 10µm. **b**, Live imaging of ACF (orange arrows) at different times of the experiment in Apc;N1CreERT2 mice subjected to 1 month permanent mechanical pressure with or without Vande treatment. **c**, Quantitative counting of ≥4 pRet+ cells per crypt signal after 1 month of *in vivo* permanent mechanical pressure in presence or absence of Vande gavage, n=6-9 mice for each condition. N= 2 experiments. Mann-Whitney test; *0.05 and ***p<0.001. **d**, ACF number counting at different times until 1 month of permanent pressure on the colon of Apc;N1CreERT2 mice with or without Vande treatment, n=7-9 mice/condition. Statistical significance determined using the Holm-Sidak method, with alpha = 0.05. Adjusted p (D28, UMLm vande vs. UMLm veh) <0.05. Error bars: standard deviation, except for d in which it is standard error to the mean.

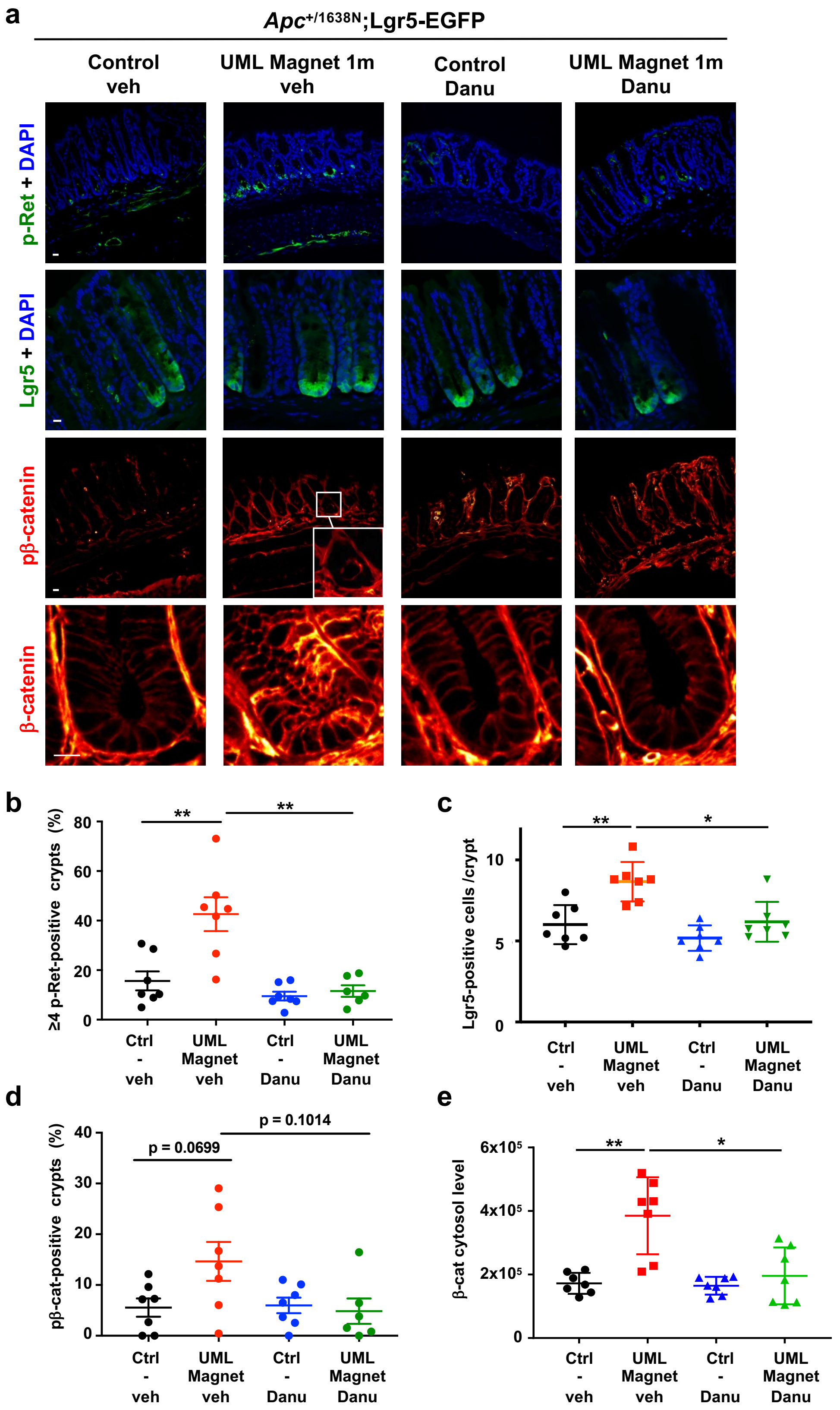

Supplementary Figure 17

Supplementary Figure 17. Pharmacological inhibition of the Ret/β-cat pathway with the pRet inhibitor Danu in Apc;Lgr5-EGFP mice. a, Levels of pRet+, Lgr5+ SC, p-βcat+ and β-cat+ crypts in Apc;Lgr5-EGFP mice after application of a permanent magnetic compression mimicking tumor growth

pressure for 1 month, with and without Danu. Control veh: mice without UML injection treated for 1 month with vehicle of Danu (n=7 mice); UML+Magnet 1m veh: mice injected with UML plus permanent magnet implantation and treated with vehicle for 1 month (n=7 mice); Control Danu: mice without UML injection treated for 1 month with Danu (n=7 mice); UML+Magnet 1m Danu: mice injected with UML plus permanent magnet implantation and treated with Danu for 1 month (n=7 mice). N=2 experiments. Scale bar is 10µm. **b**, Quantitative analysis of the pRet signal. Percentage of crypts with ≥4 pRet positive cells per mouse. **c**, Quantitative analysis of Lgr5-EGFP signal. Mean number of Lgr5+ SC per crypt and per mouse. **d**, Quantitative analysis of the pY654 β-cat signal. Percentage of pY654 β-cat positive crypts per mouse. **e**, Quantitative analysis of the β-cat signal. The values represent the Mean Integrated Density (IntDen) signal of a minimum of 10 crypts per mouse measured with ImageJ. Mann-Whitney test; * $p<0.05$ and ** $p<0.01$. Error bars: standard deviation.

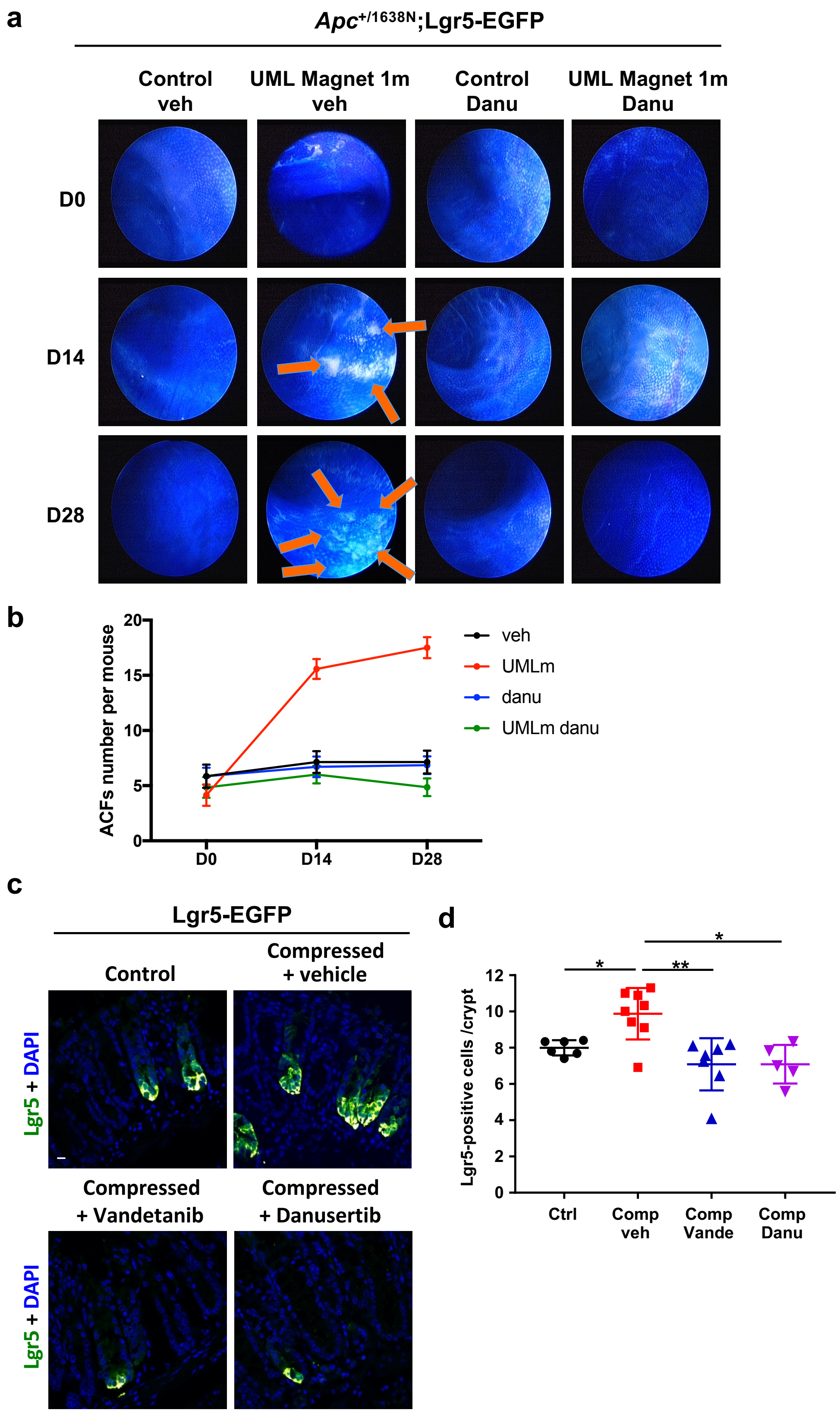

Supplementary Figure 18

Supplementary Figure 18. The pharmacological inhibition of the Ret/β-cat pathway with Danu inhibits mechanical induction of ACFs. a, Live imaging of ACFs (orange narrow) at different times of

the experiment subjected to the application of a permanent magnetic compression mimicking tumor growth pressure for 1 month, with or without Danu treatment in Apc;Lgr5-EGFP mice. **b**, ACFs number counting at different times until 28 days of the application of permanent magnetic compression mimicking tumor growth pressure application on the colon of Apc;Lgr5-EGFP mice with or without Danu treatment, n=7 mice/condition. N=2 experiments. Statistical significance determined using the Holm-Sidak method, with alpha=0.05. **c**. The mechanical stimulation of SC is pRet dependent in Lgr5-EGFP mice colon, *ex vivo.* Levels of Lgr5+ SC observed after 20 min of 1kPa *ex vivo* mechanical compression and 4 hours incubation on Lgr5-EGFP mice colon explants. Scale bar is 10µm. **d.** Quantification of Lgr5-EGFP signal after 20 min of 1 KPa compression and 4 hours incubation with vehicle, Vande or Danu. Mean number of Lgr5+ SC per crypt and per mouse. Scale bar is 50µm. Mann-Whitney test; * $p<0.05$; ** $p<0.01$. Error bars: standard deviation, except for b in which it is standard error to the mean.

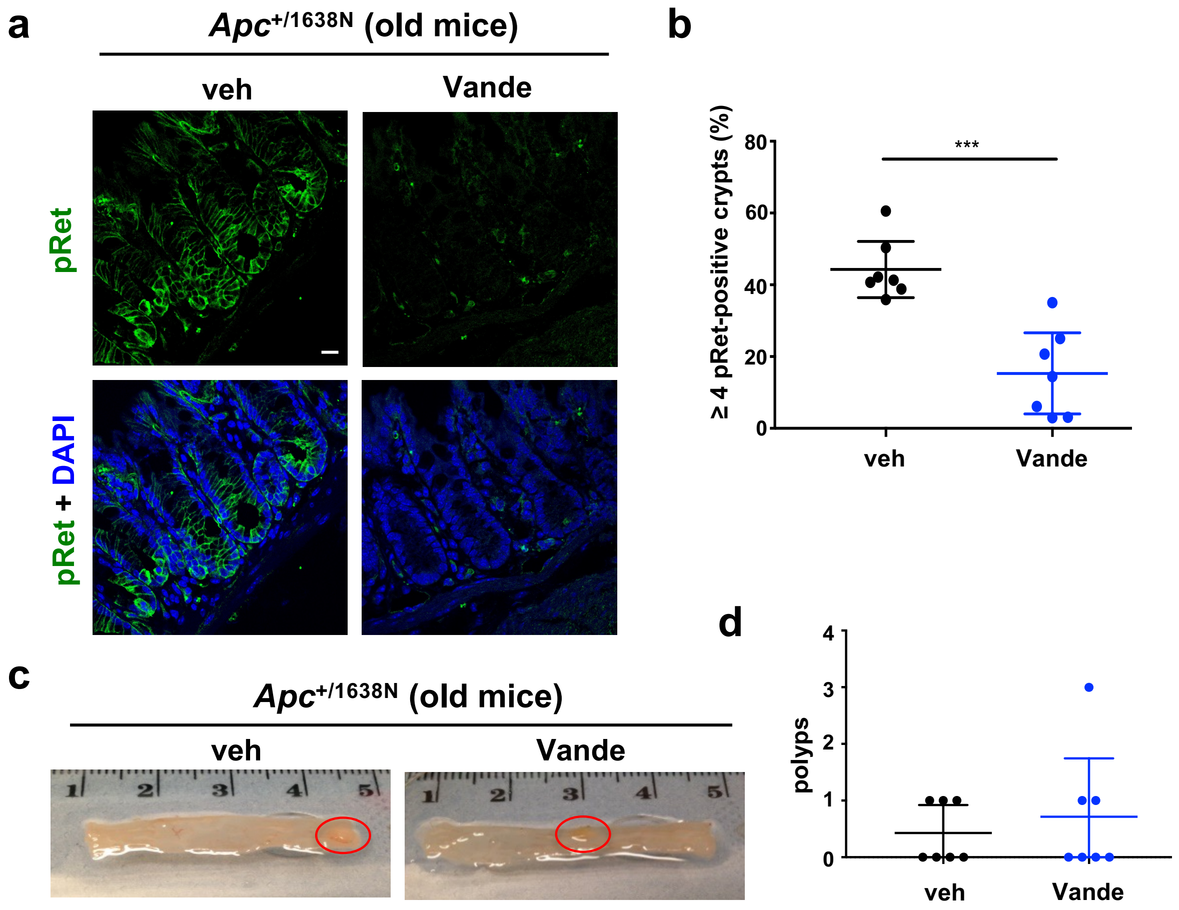

Supplementary Figure 19

Supplementary Figure 19. The pharmacological inhibition of the Ret/β-cat pathway inhibits the endogenous increase in ACFs in old predisposed Apc mice. a, Levels of Ret phosphorylation in old Apc mice colon after 1 month of vehicle or Vande treatment. Scale bar is 10µm. **b**, Quantitative counting of pRet+ crypts after 1 month of vehicle or Vande treatment, n=7 mice for each condition. Statistical significance determined using the Mann-Whitney test; ***p<0.001. **c**, Distal colon pictures after 1 month of vehicle or Vande treatment of Apc old mice, n=7 mice for each condition. Polyps are surrounded by a red circle. **d**, Polyps counting after 1 month of vehicle or Vande treatment. Note that most of polyps are already present before treatment. n=7 mice/condition. Mann-Whitney test; ns: not significant. Error bars: standard deviation.

Supplementary References

1 Roberts, R. R., Bornstein, J. C., Bergner, A. J. & Young, H. M. Disturbances of colonic motility in mouse models of Hirschsprung's disease. *Am J Physiol Gastrointest Liver Physiol* **294**, G996-G1008, doi:10.1152/ajpgi.00558.2007 (2008).

2 Mancinelli, R. *et al.* Inhibition of peristaltic activity by cannabinoids in the isolated distal colon of mouse. *Life sciences* **69**, 101-111, doi:10.1016/s0024-3205(01)01110-9 (2001).

3 Spencer, N. J., Dinning, P. G., Brookes, S. J. & Costa, M. Insights into the mechanisms underlying colonic motor patterns. *The Journal of physiology* **594**, 4099-4116, doi:10.1113/JP271919 (2016).

4 Fernandez-Sanchez, M. E. *et al.* Mechanical induction of the tumorigenic beta-catenin pathway by tumour growth pressure. *Nature* **523**, 92-95, doi:10.1038/nature14329 (2015).

5 Thebault, C. J. *et al.* In Vivo Evaluation of Magnetic Targeting in Mice Colon Tumors with Ultra-Magnetic Liposomes Monitored by MRI. *Mol Imaging Biol* **21**, 269-278, doi:10.1007/s11307-018-1238-3 (2019).

6 Bealle, G. *et al.* Ultra Magnetic Liposomes for MR Imaging, Targeting, and Hyperthermia. *Langmuir : the ACS journal of surfaces and colloids* **28**, 11834-11842, doi:10.1021/la3024716 (2012).

7 Parish, I. A. *et al.* A Novel Mutation in Nucleoporin 35 Causes Murine Degenerative Colonic Smooth Muscle Myopathy. *Am J Pathol* **186**, 2254-2261, doi:10.1016/j.ajpath.2016.04.016 (2016).

8 Ryan, A. J. & Wedge, S. R. ZD6474--a novel inhibitor of VEGFR and EGFR tyrosine kinase activity. *British journal of cancer* **92 Suppl 1**, S6-13, doi:10.1038/sj.bjc.6602603 (2005).

9 Roper, J. C. *et al.* The major beta-catenin/E-cadherin junctional binding site is a primary molecular mechano-transductor of differentiation in vivo. *eLife* **7**, doi:10.7554/eLife.33381 (2018).

10 Peifer, M. & Wieschaus, E. The segment polarity gene armadillo encodes a functionally modular protein that is the Drosophila homolog of human plakoglobin. *Cell* **63**, 1167-1176. (1990).

11 Serrano, C. *et al.* Compartmentalized Response of IL-6/STAT3 Signaling in the Colonic Mucosa Mediates Colitis Development. *J Immunol* **202**, 1239-1249, doi:10.4049/jimmunol.1801060 (2019).

12 Whitehead, J. *et al.* Mechanical factors activate beta-catenin-dependent oncogene expression in APC mouse colon. *HFSP journal* **2**, 286-294, doi:10.2976/1.2955566 (2008).

13 Wang, D. *et al.* Paneth cell marker expression in intestinal villi and colon crypts characterizes dietary induced risk for mouse sporadic intestinal cancer. *Proc Natl Acad Sci U S A* **108**, 10272-10277, doi:10.1073/pnas.1017668108 (2011).

14 Huels, D. J. *et al.* Wnt ligands influence tumour initiation by controlling the number of intestinal stem cells. *Nature communications* **9**, 1132, doi:10.1038/s41467-018-03426-2 (2018).
