# Supplementary material for: Ret kinase-mediated mechanical induction of colon stem cells by tumor growth pressure stimulates cancer progression in vivo
Source: Commun Biol. 2022 Feb 17;5:137. doi: 10.1038/s42003-022-03079-4 (PMC8854631; doi:10.1038/s42003-022-03079-4)
Supplement: Supplementary file 3 — Description of Additional Supplementary Files [file 42003_2022_3079_MOESM3_ESM.pdf]

Description of Additional Supplementary Files

File name: Supplementary Movie 1
Description: Visualization of endogenous colonic pulsatile waves using ultrasound in vivo: before WIN.

File name: Supplementary Movie 2
Description: Visualization of endogenous colonic pulsatile waves using ultrasound in vivo: after WIN.

File name: Supplementary Movie 3
Description: Set-up to induce a pulsated magnetic field gradient mimicking colonic high frequency pulsatile stresses.

File name: Supplementary Movie 4
Description: Visualization of endogenous colonic pulsatile waves using ultrasound in colon loaded with UML ex-vivo.

File name: Supplementary Movie 5
Description: Visualization of endogenous colonic pulsatile waves using ultrasound in colon loaded with UML and periodic magnetic field leading to 1kPa pulsed pressure ex-vivo.

File name: Supplementary Data 1
Description: Source data of manuscript principal and supplementary figures
