# Supplementary material for: Ret kinase-mediated mechanical induction of colon stem cells by tumor growth pressure stimulates cancer progression in vivo
Source: Commun Biol. 2022 Feb 17;5:137. doi: 10.1038/s42003-022-03079-4 (PMC8854631; doi:10.1038/s42003-022-03079-4)
Supplement: Supplementary file 10 — Reporting Summary [file 42003_2022_3079_MOESM10_ESM.pdf]

nature research

Corresponding author(s): Emmanuel FARGE

Last updated by author(s): Jan 12 2022

Reporting Summary

Nature Research wishes to improve the reproducibility of the work that we publish. This form provides structure for consistency and transparency in reporting. For further information on Nature Research policies, see our Editorial Policies and the Editorial Policy Checklist.

Statistics

For all statistical analyses, confirm that the following items are present in the figure legend, table legend, main text, or Methods section.

n/a	Confirmed	
☐	☒	The exact sample size (*n*) for each experimental group/condition, given as a discrete number and unit of measurement
☐	☒	A statement on whether measurements were taken from distinct samples or whether the same sample was measured repeatedly
☐	☒	The statistical test(s) used AND whether they are one- or two-sided<br>*Only common tests should be described solely by name; describe more complex techniques in the Methods section.*
☒	☐	A description of all covariates tested
☒	☐	A description of any assumptions or corrections, such as tests of normality and adjustment for multiple comparisons
☐	☒	A full description of the statistical parameters including central tendency (e.g. means) or other basic estimates (e.g. regression coefficient) AND variation (e.g. standard deviation) or associated estimates of uncertainty (e.g. confidence intervals)
☒	☐	For null hypothesis testing, the test statistic (e.g. *F*, *t*, *r*) with confidence intervals, effect sizes, degrees of freedom and *P* value noted<br>*Give P values as exact values whenever suitable.*
☒	☐	For Bayesian analysis, information on the choice of priors and Markov chain Monte Carlo settings
☒	☐	For hierarchical and complex designs, identification of the appropriate level for tests and full reporting of outcomes
☒	☐	Estimates of effect sizes (e.g. Cohen's *d*, Pearson's *r*), indicating how they were calculated

Our web collection on statistics for biologists contains articles on many of the points above.

Software and code

Policy information about availability of computer code

Data collection	NA
Data analysis	NA

For manuscripts utilizing custom algorithms or software that are central to the research but not yet described in published literature, software must be made available to editors and reviewers. We strongly encourage code deposition in a community repository (e.g. GitHub). See the Nature Research guidelines for submitting code & software for further information.

Data

Policy information about availability of data

All manuscripts must include a data availability statement. This statement should provide the following information, where applicable:

- Accession codes, unique identifiers, or web links for publicly available datasets
- A list of figures that have associated raw data
- A description of any restrictions on data availability

The data sets generated and analysed during the current study are available from the corresponding authors on reasonable request. This information is provided in the manuscript, with the list of figures that have associated raw data.

Field-specific reporting

Please select the one below that is the best fit for your research. If you are not sure, read the appropriate sections before making your selection.

☒ Life sciences ☐ Behavioural & social sciences ☐ Ecological, evolutionary & environmental sciences

For a reference copy of the document with all sections, see nature.com/documents/nr-reporting-summary-flat.pdf

Life sciences study design

All studies must disclose on these points even when the disclosure is negative.

Sample size	Sample size was empirically increased until an initial tendancy in the expermements could, be confirmed by a p-value <0.05. Experiments were generally stopped around 10 sample by condition maximum, whatever the result.
Data exclusions	NA
Replication	All experiments were replicated independently at least 2 times.
Randomization	No randomization was performed. Many distinct experiments were performed basically addressing the same concept with different controle parameters, overall de facto excluding covariates (7 figures, 19 supplementary Figures) (for instance blocking mechanical strains by WIN (parameter pharmacological in nature), and rescuying it with magnetic forces (parameter physical in nature)).
Blinding	No blinding was performed. The many experiments realized in this study made it highly complex to manage. However, experiments were analysed in parallel by two independent investigators on the different elements of the pathway to check for convergence of the results.

Reporting for specific materials, systems and methods

We require information from authors about some types of materials, experimental systems and methods used in many studies. Here, indicate whether each material, system or method listed is relevant to your study. If you are not sure if a list item applies to your research, read the appropriate section before selecting a response.

Materials & experimental systems

n/a	Involved in the study	
☐	☒	Antibodies
☒	☐	Eukaryotic cell lines
☒	☐	Palaeontology and archaeology
☐	☒	Animals and other organisms
☒	☐	Human research participants
☐	☒	Clinical data
☒	☐	Dual use research of concern

Methods

n/a	Involved in the study	
☒	☐	ChIP-seq
☒	☐	Flow cytometry
☒	☐	MRI-based neuroimaging

Antibodies

Antibodies used	These are decribed into the manuscript and introduced in a dedicated table in the Suplementary Information section.
Validation	All antibodies were validated by publications or providers, and cited by the provider associated to the reference introduced in the up-cited table.

Animals and other organisms

Policy information about studies involving animals; ARRIVE guidelines recommended for reporting animal research

Laboratory animals	Apc+/1638N , Lgr5-EGFP-ires-creERT2 , Notch1-CreERT2/Rosa26mTmG , double transgenic Notch1-CreERT2/Rosa26mTmG, Apc +/1638N;Lgr5-EGFP-ires-creERT2 and Apc+/1638N;Notch1-CreERT2/Rosa26mTmG , C57Bl/6J . This information can be found in the Method section.
Wild animals	NA
Field-collected samples	NA
Ethics oversight	Experimental procedures were specifically approved by the ethics committee of the Institut Curie CEEA-IC #118 (Authorization reference APAFIS#10977-201708211557193 v3 given by the national authority French Ministry of Research in compliance with the international guidelines

Human Biopsies: Clinic French Ethical Committee (Agreement number D-750602, France) and the ethics committee of the Institut Curie (Agreement number C75-05-18).

Note that full information on the approval of the study protocol must also be provided in the manuscript.

Clinical data

Policy information about clinical studies

All manuscripts should comply with the ICMJE guidelines for publication of clinical research and a completed CONSORT checklist must be included with all submissions.

Clinical trial registration	NA
Study protocol	NA
Data collection	Extensive library of human colon, and other organs, cancer biopsies of the pathology service of the Institut Curie hospital, were used. These were obtained at Curie Institute and from multi-center Departments of Pathology from 1978 to 2010. For each type of tumor, ten specimens of adjacent normal tissue were used as control at RNA and protein levels. Patients treated in our institution have given their approval by signed informed consent. All patients all met the following criteria: primary tumor for which complete clinical, histologic, and biologic data were available. Quality of the data managagement is ensured by CDT labelisation of the Institut Curie by the Inca. This can be found into the manscuript.
Outcomes	The outcome tested was the activation of the mechanosenstive Ret phosphorylation in samples, monitored by specific phospho-antibodies y. Biopsies cross-cuts were classically generated and labelled by the Institut Curie Pathex, with a level of labelling semi-quantitatively evaluated from 0,1,2 to 3. Statistical analysis were performed based on these analysis by comparison with WT samples, with a p-value being addressed to any element of the pathway tested in any type of tumour and grade tested.
